# A reliability analysis method for Christmas tree in operating conditions based on FEMCA, FTA and numerical simulation

**DOI:** 10.1371/journal.pone.0359208

**Published:** 2026-10-08

**Authors:** Qiong Wang, Huimin Li, Guanlong Ren

**Affiliations:** 1 School of Architecture and Engineering, Zhanjiang University of Science and Technology, Zhanjiang, Guangdong, China; 2 School of Education Management, Assumption University, Bangkok, Thailand; 3 School of Management, Xi’an University of Architecture and Technology, Xi’an, Shaanxi, China; 4 CNOOC Zhanjiang Branch Company, Zhanjiang, Guandong, China; University of Catania, ITALY

## Abstract

The installation and recovery process of Christmas tree is complex and technically difficult, and it bears high internal and external loads during production and has extremely high requirements for operation reliability. To ensure the safe operation of China’s first domestically produced deepwater subsea Christmas tree installation and production conditions, a reliability analysis method for Christmas tree in operating conditions based on FEMCA, FTA and numerical simulation is established. Firstly, the FEMCA method is used to analyze the detailed operation steps of drill pipe installation method. The severity and hazard degree of the failure modes of each component during the installation and recovery process of Christmas tree are determined. The main failure modes are evaluated as deformation of the tree body, leakage of valve groups, leakage or blockage of connecting pipe fittings. Secondly, using the FTA method and based on the OREDA database, statistical analysis is conducted on the failure data and related failure modes. A fault tree model is established for 11 main component structures during the production operation, and the basic event probability, importance, and ranking of each component structure are calculated. The results shows that the average failure probability during production is 25.82 times per 10^6^ hours, and its reliability parameters has an average failure interval of 4.42 years. The overall reliability level meets the production safety requirements. Thirdly, the stability numerical simulation analysis shows that the maximum stress ratio of the Christmas tree system under different ocean current conditions is 0.529, and the maximum bending moment is 7292 kN·m, and the safety operation window is established with the overall reliability level of the system meeting production safety requirements.

## 1. Introduction

The subsea Christmas tree is the core component of the subsea oil and gas production system, and also the basis for building the subsea oil and gas well production system. Its main function is to control the flow of oil and gas from the wells, and it can also be used to control the injection of gas or water into non production wells. Due to the complex structure and high quality of the Christmas tree system, its design, manufacturing and installation operations are extremely difficult. In addition, the complex and changing working environment on the sea and the increasing water depth in future operations. Firstly, during the installation process of Christmas tree it may be damaged due to strong nonlinear environmental loads, ship movements, human factors and other reasons. In severe cases, it may endanger the installation and construction operations and cause major disasters such as installation overturning or later production leakage [1,2]. Secondly, the Christmas tree is connected to the subsea wellhead and shallow wellbore during its operational production phase, involving complex internal processes such as high-temperature and high-pressure oil and gas production, chemical reagent injection, and subsea control system power/communication for extended periods. Each component has a dual effect of internal production and external environmental loads. When the load borne by the Christmas tree exceeds the design limit, major safety accidents such as valve failure, pipeline erosion and even oil and gas leakage will occur [3,4]. Therefore, the operating risk of subsea Christmas tree during installation and operation is high and to improve the operation safety and reliability of the Christmas tree is crucial.

The safety risk analysis is to select appropriate analysis methods for key components or operating processes of the Christmas tree, and obtain the risk factors, failure probability and risk impact of corresponding components at the system level, and in order to combine corresponding measures and suggestions to improve reliability. According to the purpose of risk analysis, available reliable data and decision-making needs, risk assessment methods are mainly divided into three categories: qualitative, semi quantitative and quantitative. At present, there are many risk analysis methods used for the installation and recovery process, the installation process of tubing hanger, the sea trial process, and the flow safety guarantee of overflow channels of the Christmas tree. The main qualitative methods include Fault Tree Analysis (FTA), Failure Mode, Effects and Criticality Analysis (FMECA), Analytic Hierarchy Process (AHP), Hazard and Operability Analysis (HAZOP), and quantitative methods such as Technique for Order Preference by Similarity to Ideal Solution(TOPSIS), Bayesian Network (BN), and Mechanical Modeling Analysis(MMA), etc [5]. DUAN and Luo et al. [6] conducted a risk analysis during the installation process of horizontal Christmas trees using the fuzzy fault tree analysis method. Based on the qualitative analysis of the fault tree, they established the AHP-TOPSIS comprehensive evaluation method to quantitatively assess the multiple risk factors during the installation and leakage process of Christmas tree. Xiao et al. [7] used the AHP method to construct an evaluation index system for Christmas tree sea trials, and used fuzzy analysis method to conduct risk assessment on the comprehensive performance of Christmas tree sea trials. A. S. Cheliyan and Liu et al. [8,9] conducted a risk analysis of oil and gas leakage in Christmas tree system using a combination of fault trees and Bayesian networks. Wang et al. [5] addressed the shortcomings in the reliability analysis of Christmas tree and analyzed the applicability of four typical risk assessment methods for shallow water subsea Christmas tree. They also proposed practical engineering recommendations for shallow water subsea Christmas tree. Ping et al. used the Layer of Protection Analysis (LOPA) method to semi quantitatively analyze the Safety Integrity Level (SIL) classification of typical Christmas tree processes, which ensuring the safe and efficient operation of the subsea production system. Zhu et al. [10] used the fuzzy fault tree analysis method to conduct reliability analysis on the Christmas tree system, and further studied the failure mechanism of the Christmas tree. They established a Markov model for the Christmas tree system and quantitatively analyzed its reliability. Liu et al. [8] analyzed the system reliability of the Christmas tree during operation using a combination of FTA and FMEA methods. Det Norske Veritas has developed a series of assessment software for offshore oil engineering, mainly including Neptune Offshore® for risk assessment and hazard assessment in offshore oil and gas development equipment, Leak® for calculating the frequency of various petrochemical plant accidents, and SAFETI® for conducting multifunctional risk assessment and hazard assessment [11,12].

In general, scholars at China and abroad have conducted extensive analysis on the reliability of Christmas tree under typical operating conditions, involving various reliability analysis methods. However, there has been no use combined with FEMCA and FTA method to conduct risk analysis during the installation and recovery process of the Christmas tree, nor has there been a specific calculation of the failure probability and safety risk assessment of various components of the Christmas tree during operation conditions. And there is a lack of comprehensive risk analysis method from system to components of the Christmas tree in operating conditions [13,14]. Therefore, based on previous research and combined with the operational experience and installation resources in the South China Sea, firstly, this article uses the FMECA analysis method to conduct a risk analysis for the installation and recovery process of China’s first domestically produced deepwater Christmas tree, and the main failure modes are evaluated. Secondly, the FTA fault tree analysis method is used to calculate the failure probability of each major component of the Christmas tree system combined with the system failure statistical data of the in-service working conditions of the Christmas tree. Thirdly, numerical simulation analysis methods are used to analyze the stability of the system composed of Christmas tree and wellhead under different operating conditions, ultimately forming an a reliability analysis method based on FEMCA, FTA and numerical simulation. Corresponding suggestions and measures are proposed using the Bow model, which can provide technical support for the safe installation and recovery of the Christmas tree, as well as safe operation during production.

## 2. Operating conditions analysis

### 2.1 Christmas tree

The Christmas tree is the core component of subsea oil and gas production equipment, and is also the foundation for building subsea oil and gas production system. It mainly consists of the tree body, tubing hanger, valves, pipelines and wellhead connectors. The oil and gas flow through the wellhead, tubing hanger, production channel, throttle valve, and then transport to the subsea production manifold (Fig 1).

**Fig 1:**
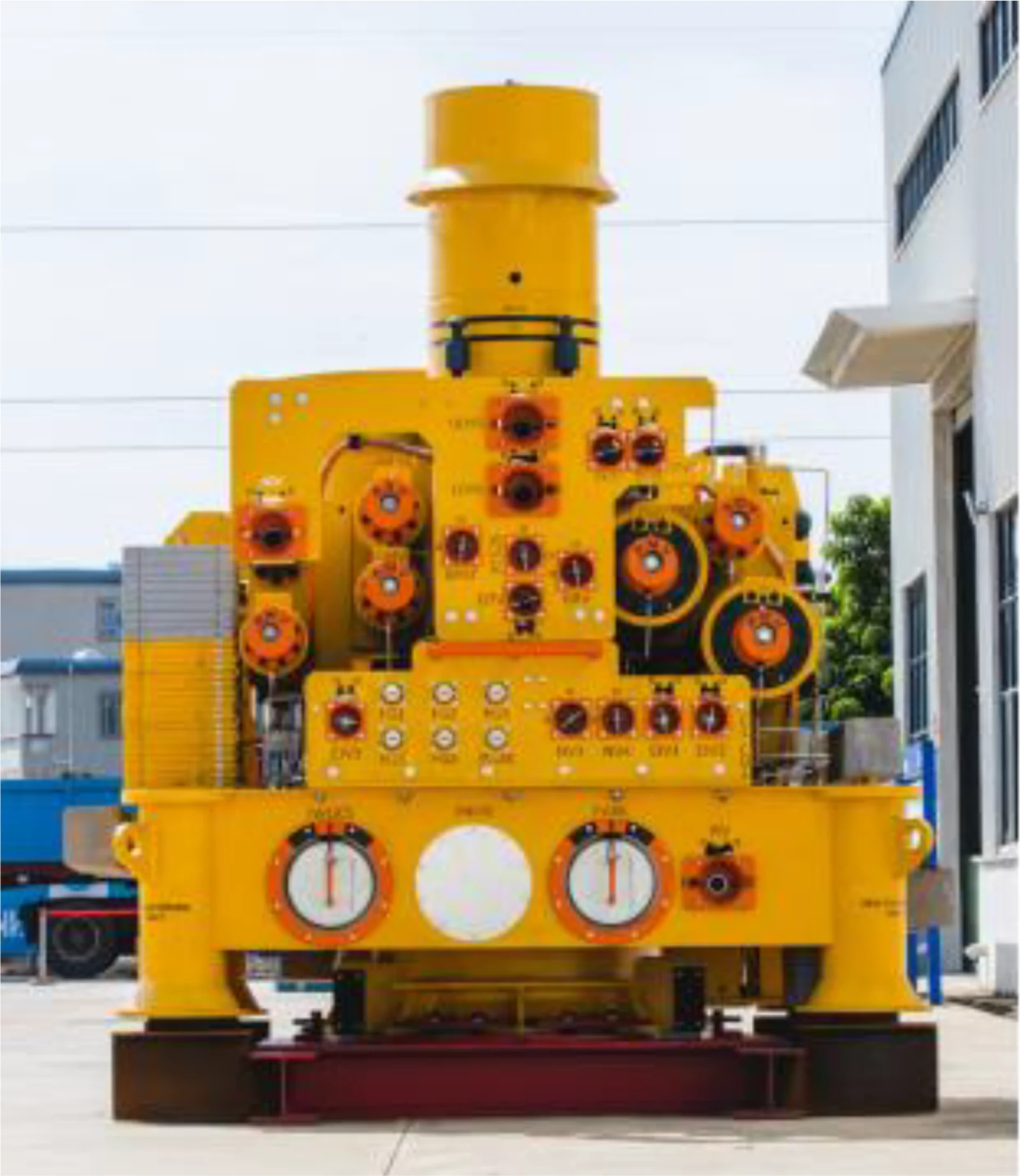
A horizontal Christmas tree. It is the China’s first domestically produced deepwater Chirstmas tree. It is 9 meters long, 8.6 meters wide, and 6.5 meters high, with a total of 2,500 parts and a weight of 55 tons. It has passed dual certification from DNV and CCS. It has been successfully applied in the South China Sea.

### 2.2 Installation condition

The installation of the Christmas tree is a complex technical operation, which involves many risk points. At present, the installation and recovery methods of Christmas tree at China and abroad mainly include wire rope method, drill pipe installation method, pulley lowering method, etc. The advantages and disadvantages of each method are shown in Table 1. Based on the analysis of the advantages and disadvantages of various methods mentioned above, and considering China’s experience in South China Sea operations and installation resources, it is recommended to use the drill pipe installation method for the installation of the China’s first domestically produced deepwater Christmas tree.

**Table 1:** Comparison and analysis of main installation methods for Christmas tree.

Method	Applicable water depth	Advantage	Disadvantage	Difficulty
Drill pipe installation method	< 1500m	Low installation cost. Easy installation and operation.The installation technology is mature.	Lower installation is limited by water depth. The installation of climate windows requires relatively high standards. We need a heave compensation device.	General
Pulley lowering method	< 3000m	High carrying capacity. The cable experiences less force.	Need to modify the drilling rig.There is a risk of resonance at a certain depth. Two operational vessels are required, resulting in high costs.	Higher
Lower swing method	< 3000m	The installation ship has high operational adaptability and no specific requirements for the installation ship. The installation of climate windows requires high standards.	The installation is relatively complex, requiring two operating vessels, high cost, and certain requirements for the lowered sea area. The positioning accuracy is not high.	Higher
Pencil float method	< 3000m	Low operational risk, unrestricted installation equipment size, high applicability to installation vessels, and low requirements for installing climate windows.	High installation cost, and deepwater installation relies heavily on the winch capacity. Only one subsea equipment can be installed in one towing operation, resulting in low installation efficiency.	Higher
Moon pool wet dragging method	< 3000m	Avoid operating through splash zones, have low requirements for installing climate windows, and have high applicability for installation ships.	Wet towing has high costs and requires high installation capacity for ship winches. The installation ship needs to have heave compensation function.	Higher

Drill pipe installation method is a commonly used method for subsea equipment installation on drilling platforms, mainly using the drilling platform and drill pipes for auxiliary lowering [15]. The general steps are to first transport the Christmas tree from a barge without self propulsion capability to a designated location close to the drilling platform, then use the main crane of the drilling platform to lift the Christmas tree from the transport barge to the drilling platform, and then adjust it to the transport slide rail and move it to the moon pool. Then, use drill pipes to lower the equipment to the seabed for installation. Specifically, it includes: (1) conduct pre installation testing of the Christmas tree. (2) recycle the temporary abandoned well caps. (3) connect the Christmas tree to lowering tool. (4) drill pipe lowering, ROV auxiliary observation. (5) lock the Christmas tree to the subsea wellhead. (6) connect the Christmas tree and install the umbilical cable. (7) use IWOCS for final testing of the Christmas tree. (8) install the protection cover (Fig 2).

**Fig 2:**
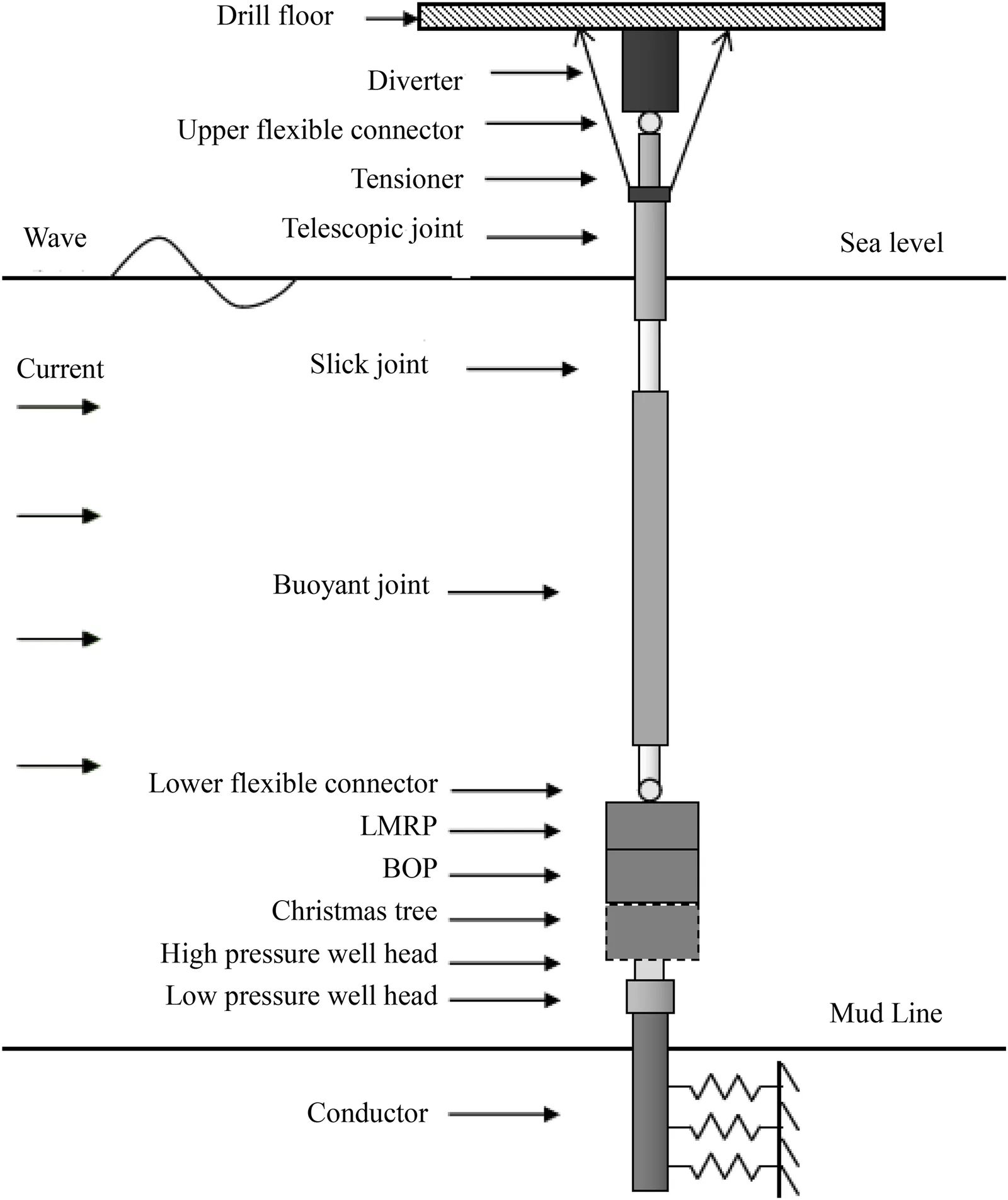
Installation condition structure diagram of the Christmas tree. It is a schematic diagram of the subsea equipment after the installation with the drill pipe. It primarily consists of the drilling platform, riser, BOP, Christmas tree, and subsea wellhead, arranged from top to bottom. The Christmas tree is positioned between the BOP and the subsea wellhead, and is connected to them by H4 hydraulic connectors.

### 2.3 Recycle condition

When the Christmas tree completes sea trials or needs to be repaired or abandoned during the production of oil and gas wells, the Christmas tree is recovered [16]. The recovery of the tree also adopts the drill pipe method, which specifically includes: (1) cleaning the fishing nets and marine organisms covered by the Christmas tree. (2) lower the drill pipe to retrieve the Christmas tree protective cover. (3) lower the drill pipe to retrieve the high-pressure outer cap of the Christmas tree. (4) lower the drill pipe to recover the protective leg of the Christmas tree. (5) unlocking the Christmas tree and subsea wellhead. (6) remove the umbilical cable of the Christmas tree. (7) connect the Christmas tree to recovery tool, lift and lower the drill pipe, and use ROV auxiliary observation.

### 2.4 Operation condition

#### 2.4.1 Load analysis of the Christmas tree in operation condition.

During the normal production process of the Christmas trees, due to the higher temperature of the reservoir oil and gas than the surrounding environment, heat is continuously dissipated to the main body of the Christmas tree under the action of radial temperature difference. Under the pressure of the formation, the main body of the Christmas tree is subjected to load changes caused by temperature and pressure. At the same time, the production channel structure of the Christmas tree is complex, and as the temperature and pressure changing, natural gas hydrates are easily formed at the internal throttle valve of the oil and gas, which further increases the load and acts on the Christmas tree (Fig 3) [17]. The Christmas tree contains many valve channels inside, and the production fluid may generate local turbulence inside the tree, which may cause vortex induced vibration fatigue damage to the tree. The tubing hanger inside the Christmas tree needs to consider the stress effects of temperature and pressure changes and tubing weight under normal production conditions. At the same time, the wellhead connector connected to the Christmas tree will also generate axial separation force and mechanical load due to internal pressure. During oil and gas production, sand may be produced, and solid particles mixed in the oil and gas may cause erosion and wear on the pipeline and wall when passing through the internal oil and gas flow channels, throttle valves, etc. of the Christmas tree at a certain speed. Severe erosion may lead to problems such as leakage and reduced external pressure resistance. There are multiple electro-hydraulic crossing channels installed on the Christmas tree [18]. During the production process, it is necessary to inject hydrate inhibitors such as methanol and ethylene glycol from the outside, conduct pressure tests on the upper and lower plugs, control the downhole safety valve, and transmit downhole pressure and temperature data. These operations will have a certain impact on the deformation contact stress of the sealing interface of the Christmas tree.

**Fig 3:**
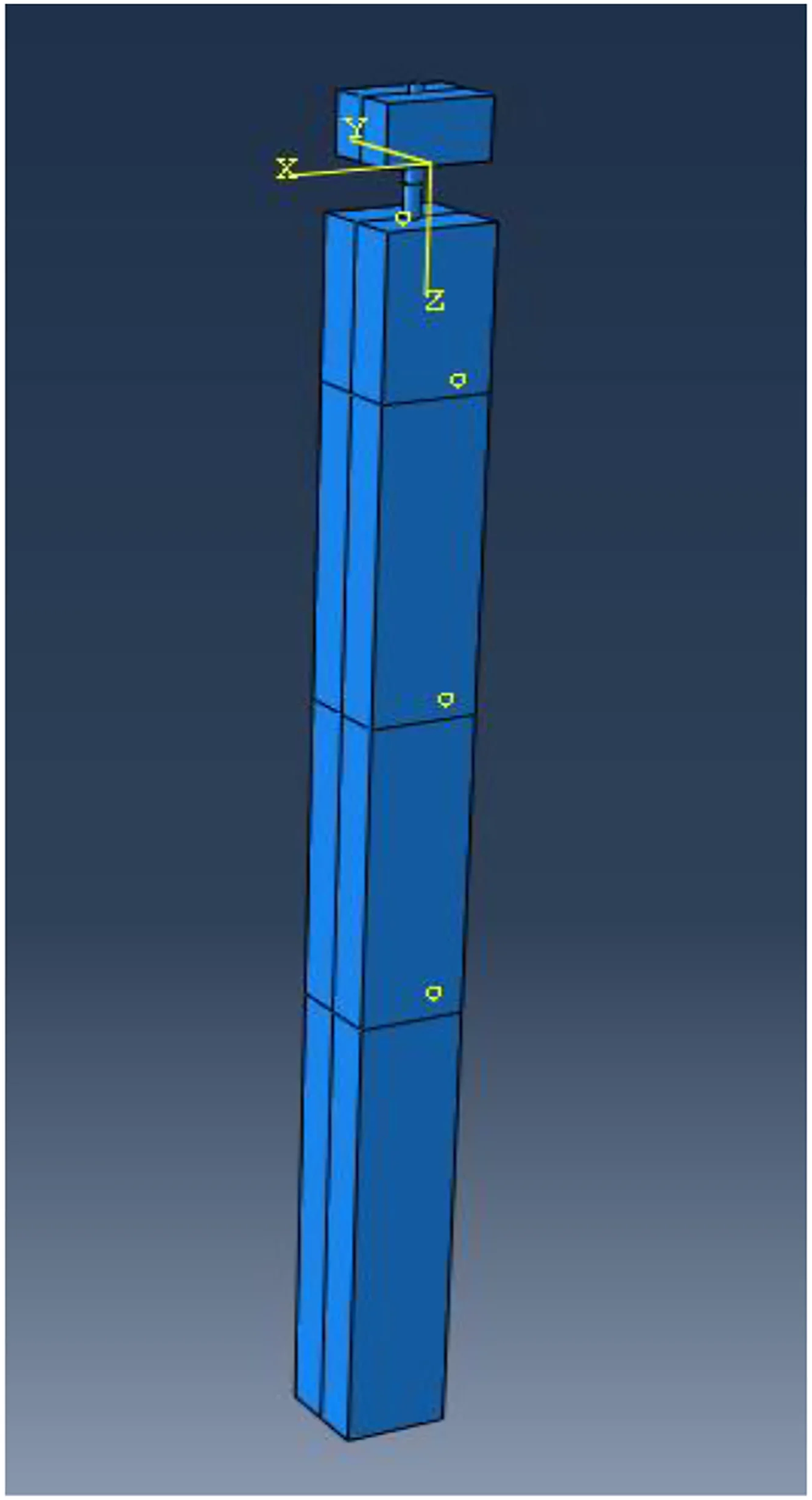
Model diagram of the Christmas tree in operation. In the model diagram, the upper blue cube represents the Christmas tree, while the lower blue rectangular prism represents the conductor located below the mudline, with the two parts connected through the subsea wellhead (thin rectangular prism) in the middle. Simply under the operation conditions, the Christmas tree is subjected to combined forces acting in the X, Y and Z directions.

#### 2.4.2 Statistical analysis of system failure in operation condition.

At present, relevant research organizations at China and abroad have conducted incomplete statistics on accidents that occur in the global production process of the Christmas tree, and have issued relevant accident reports or offshore reliability data (OREDA) based on this. Based on the OREDA database, statistical analysis has been conducted on the failure data and related failure modes of the Christmas trees. The OREDA database mainly records a series of data on the failure modes, failure frequency, failure probability, and average maintenance time of subsea production equipment such as the tree body, tree cap, top blocker, and tubing hanger, as shown in Table 2.

**Table 2:** Failure statistics of the Christmas tree subsystems during production.

Subsystem name	Failure mode	Failure reason	Maximum failure frequency (times)	Average effective repair time (H)
Hydraulic connector	External leakage Unlock malfunction	Metal sealing ring failure Sealing installation failure Hydraulic component failure Hydraulic oil leakage	2.35	11.8
Throttle module	Locking malfunction External leakage Control malfunction Block	Hydraulic component failure hydraulic oil leakage Seal aging, damage, valve core detachment, wear control signal error Tight position stuck, fully open position stuck, middle position stuck	5.85	16.875
Christmas tree body	External leakage Internal leakage Hydraulic oil leakage isolation failure Block	Seal aging, damaged seal aging, damaged seal aging, damaged seal aging, failure Hydraulic oil pollution Hydrate formation	38.56	29.1
Tubing hanger	External leakage Internal leakage	Hydraulic oil leakage, external leakage of production fluid, internal leakage of production fluid	1.51	168.0
Others	Block Corrosion Other	Leakage of choke/kill manifold Sealing failure of Christmas tree cap Corrosion damage to tree structure	1.22	31.1

From Table 2, it can be seen that the main failure modes causing the failure of the Christmas tree include leakage, unlocking/locking failure, opening/closing failure, blockage, and corrosion. The faults of the Christmas tree system mainly include hydraulic connector faults, throttling module faults, tubing hanging faults and other system faults. Among them, according to the statistics of the average effective repair time of components in the Christmas tree system, it can be seen that the maintenance time of the tubing hanger is the longest, and there is a large gap in maintenance time between it and other components. The failure probability and average maintenance time of each part of the Christmas tree are statistically analyzed, and the statistical results are shown in S1 Fig. From S1 Fig, it can be seen that the main reasons for the failure of the Christmas tree are valves, tubing hangers, hydraulic couplings, connectors, and isolation sleeves. According to the statistics of the average maintenance time of the main components of the Christmas tree, it can be seen that the maintenance time of the tubing hanger is the longest, and there is a significant difference in maintenance time between it and other components.

## 3. Method establishment

### 3.1 Failure mode, effects, and criticality analysis (FMECA)

Failure Mode, Effects and Criticality Analysis (FMECA) is a method to improve product quality and safety by analyzing the potential failure modes of various system components and their impact on product functionality, as well as the severity of the consequences. It proposes possible preventive and improvement measures. Its function is to verify the safety and reliability of the system, determine the causes of failure modes, and evaluate the potential risks that exist in the system. Due to the complex composition of the Christmas tree system, this method can discover various defects and weak links in the Christmas tree from the perspectives of product design, process, and overall system. Therefore, for all possible failures of the Christmas tree, based on the analysis of the failure modes, the impact of each failure mode on product operation is determined, single point failures are identified, and their harmfulness is determined according to the severity and probability of occurrence of the failure mode [19,20].

#### 3.1.1 Purpose and principle of FMECA.

The purpose of FMECA analysis includes: (1) identifying harmful effects and failures on system operation, such as system termination, significant degradation of system operation, or impact on user’s safety. (2) determine the harmfulness of each failure mode related to the normal function or performance of the system, locate/mitigate the priority order of each failure mode and its related process impact. (3) improve the reliability and security of the system. (4) improve the maintainability of the system. The commonly used method for hazard analysis is the Risk Priority Number (RPN) method, which uses certain statistical methods to estimate factors such as the severity S (Severity), occurrence O (Occurrence), and detection D (Detection) of the consequences when a failure occurs, then calculates the RPN and determines whether improvement is necessary or the priority of improvement based on the RPN size, thereby reducing post event losses at a lower cost and improving system reliability.

The calculation of RPN is as follows:


$$\begin{array}{c} RPN=O\times S\times D \end{array}$$ (1)

Where *O* is the frequency of failure modes occurring within a predetermined or prescribed time period; *S* is the severity of the failure occurrence; *D* is a detectable measure and an estimated probability of identifying and eliminating failures when the system or user is affected.

These three factors are scored using a 10 point grading scale, and failure modes can be classified based on RPN values. The higher the RPN value, the lower the reliability level of the evaluated object. The probability of failure mode occurrence should be considered from the following aspects: (1) experimental data from component life. (2) available failure rate database. (3) field failure data. (4) failure data of similar products or components. The failure probability classification table is shown in Table 3.

**Table 3:** Classification of failure probability.

Grade	Failure possibility	Failure probability
10	inevitable	≥1/2
9	very high	1/3
8	very frequently	1/8
7	high	1/20
6	relatively high	1/80
5	medium	1/400
4	relatively low	1/2000
3	low	1/15,000
2	very low	1/150,000
1	almost never fails	≤1/1,500,000

#### 3.1.2 Steps of FMECA.

Firstly, based on the complexity, importance, technological maturity, progress of analysis work, and cost constraints of the system, determine the product scope for FMECA in the system. Then describe the task requirements of the system and the environmental conditions in which the system completes various tasks, analyze and clarify the functions, working methods, and working hours that the products in the system should possess when completing different tasks. Further develop and analyze criteria for determining the normal and failure of the system and its products, identify all possible failure modes for each product (or function, production factor, process flow, production equipment, etc.) in the system, analyze the impact of each possible failure mode, classify them according to the severity of these impacts, and determine the severity and cause of the failure mode impact based on the failure criteria. By using failure detection method to analyze whether there are specific detection methods for each failure mode, we can provide a basis for the system’s failure detection and isolation design. Finally, a comprehensive evaluation of each product in the system is conducted based on its probability and severity of failure, and corresponding recommendations and measures are proposed (Fig 4) [21,22].

**Fig 4:**
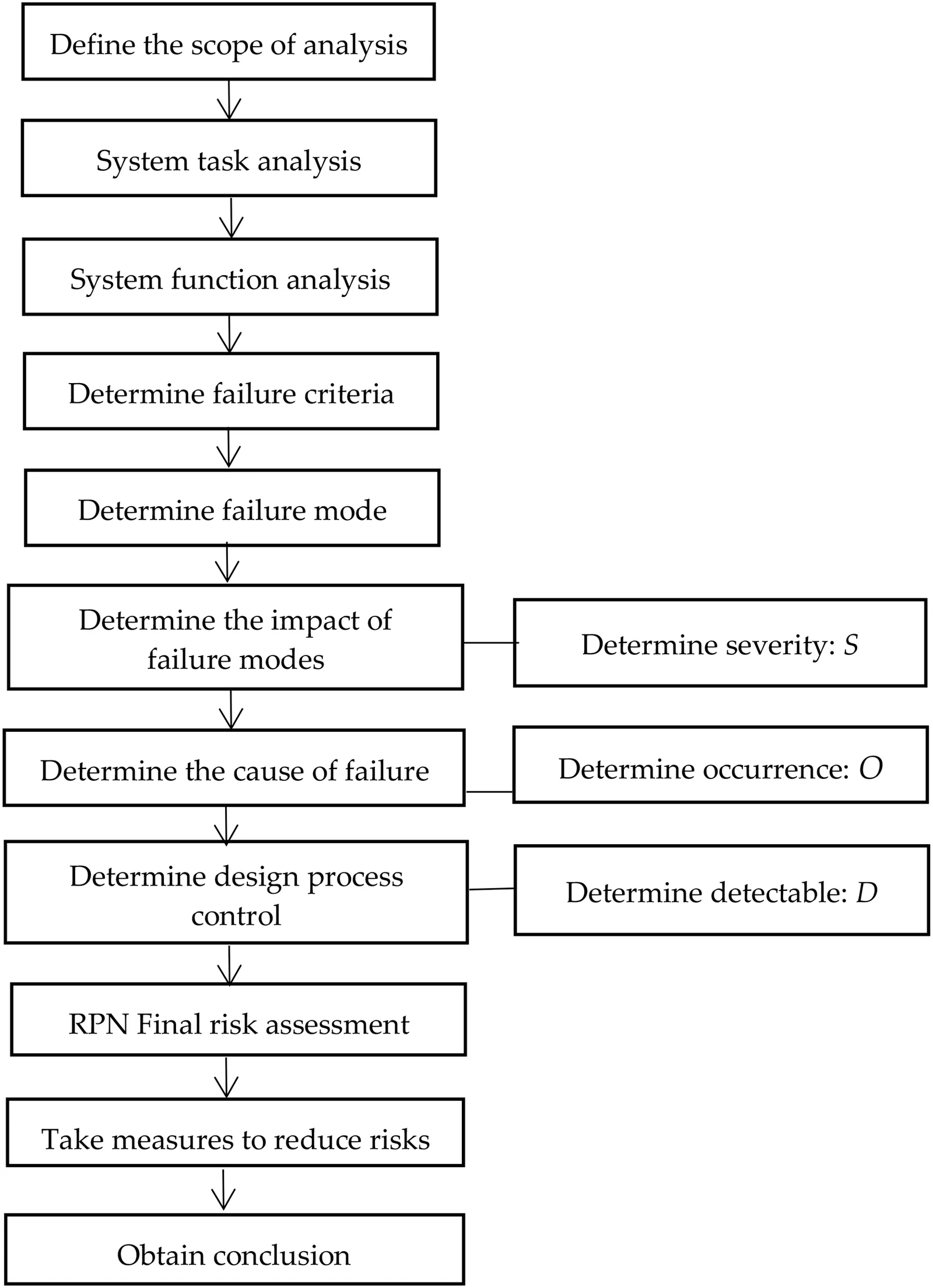
FMECA analysis flowchart. Firstly, determine the product scope. Then describe the task requirements. Further develop and analyze criteria to determine failure mode. Then by determine the S/O/D to assess the RPN risk. And then take measures to reduce the risks. Finally, a comprehensive evaluation of each product in the system is conducted.

### 3.2 Fault tree analysis (FTA)

#### 3.2.1 FTA analysis method.

Fault Tree Analysis (FTA) technology was developed by Bell Telegraph Company’s telephone laboratory in 1962. It uses logical methods to visually analyze hazards. Fault tree is a special type of inverted tree logical causal relationship diagram that describes the causal relationships between various events in a system using event symbols, logic gate symbols, and transition symbols [23,24]. There are two methods for fault tree analysis: qualitative analysis and quantitative analysis. Qualitative analysis identifies all possible fault modes that lead to the occurrence of the top event, and calculates all the minimum cut sets (MCS) of the faults. Quantitative analysis mainly involves two aspects: one is to calculate the failure probability of the system by inputting the failure probabilities of each unit (bottom event) of the system. The second step is to determine the structural importance, probability importance, and critical importance of each unit (bottom event). Finally, the optimal fault diagnosis and repair sequence can be sorted based on the magnitude of critical importance, and it can also be used as the first step to improve the data of relatively unreliable units.

This method is a “descending form” analysis approach from the system to the components, and then to the parts. It starts from the system and analyzes the probability of a fault event (also known as a top event) occurring through a branching diagram drawn by logical symbols, gradually expanding into a tree. It can also be used to analyze the impact of part, component, or subsystem failures on system failures, including human factors and environmental conditions. During the operation of the Christmas tree, various components work together. Using this method to analyze the risks and reliability of various working components during high-temperature oil and gas production has great advantages. Moreover, this method can not only qualitatively but also quantitatively analyze system failures [25]. It can not only analyze system failures caused by a single component, but also analyze system failures caused by different modes of failure of multiple components.

#### 3.2.2 Steps of FTA.

The FTA analysis steps are as follows:

(1) Familiar with Christmas tree system, define analysis objectives.
(2) Determine the top event for fault tree analysis of Christmas tree system.
(3) Identify all underlying events that caused the occurrence of the top event.
(4) Establish a fault tree and determine the minimum cut set that causes the top event to occur.
(5) Calculate the probability of the top event occurrence based on the probability of each bottom event in the fault tree.

Quantitative analysis of fault trees can be divided into solving the probability of occurrence of top events and conducting importance analysis of bottom events. When obtaining the minimum cut set *K*_1_, *K*_2_,...,*K*_R_, the probability *P*(*T*) of the top event *T* occurring is:


$$\begin{array}{c} P(T)=P({⋃}_{i=\text{1}}^{R}K_{i}) \end{array}$$ (2)

When there is no intersection between the minimum cut sets, *P*(*T*) can be solved using the following formula:


$$\begin{array}{c} P(T)=P({⋃}_{i=\text{1}}^{R}K_{i})=\sum_{i=\text{1}}^{R}P(K_{i})-\sum_{\text{1}\leq i<j\leq R}P(K_{i}K_{j})+\sum_{\text{1}\leq i<j<k\leq R}P(K_{i}K_{j}K_{k})+...+{\left(-\text{1}\right)}^{R-\text{1}}P(K_{\text{1}}K_{\text{2}...}K_{R}) \end{array}$$ (3)

When the probability of the bottom event cannot be accurately determined, the calculated probability of the top event has little significance, and when the probability of the bottom event is small, using approximate calculation can accelerate the solving speed and meet the accuracy requirements. Therefore, the following approximate formula is often used to solve the probability of the top event occurring, which is the first-order term of equation (3).


$$\begin{array}{c} P(T)\approx S_{\text{1}}=\sum_{i=\text{1}}^{R}P(K_{i}) \end{array}$$ (4)

### 3.3 Stability numerical analysis method

Under the operational production conditions of the Christmas tree, the Christmas tree is connected to the high-pressure wellhead, and the high-pressure wellhead is bound to the low-pressure wellhead. The lower part of the low-pressure wellhead is the surface conductor, which is fixed in the soil. Based on this, a coupled numerical analysis model for the overall stability of the Christmas tree under operational conditions is established. During the production period they are subjected to complex working conditions such as high temperature, high pressure, and high erosion, as well as communication processes such as hydraulic and electrical systems. Therefore, the sealing and pressure bearing requirements for Christmas tree are high, and the risk of failure is increased due to external exposure to ocean currents and corrosion in the marine environment [26]. Therefore, the correctness of FMECA and FTA risk analysis theoretical methods is verified by using stability numerical analysis methods.

The soil model is a linear elastic model and a Mohr Coulomb plastic model, which constrain soil displacement. The bottom of the pipeline constrains displacement in the X, Y, and Z directions (Fig 5). The outer surface of the conductor is in surface to surface contact with the soil, with the normal direction being a hard contact and the tangential direction being a penalty function model. Set reference points on the cross-section of the conductor at the mud surface, coupled with the cross-sections, and load seismic acceleration on the reference points. Apply ocean current loads along the y-direction to the Christmas tree and the high and low pressure wellhead, which is a uniformly distributed surface force [27].

**Fig 5:**
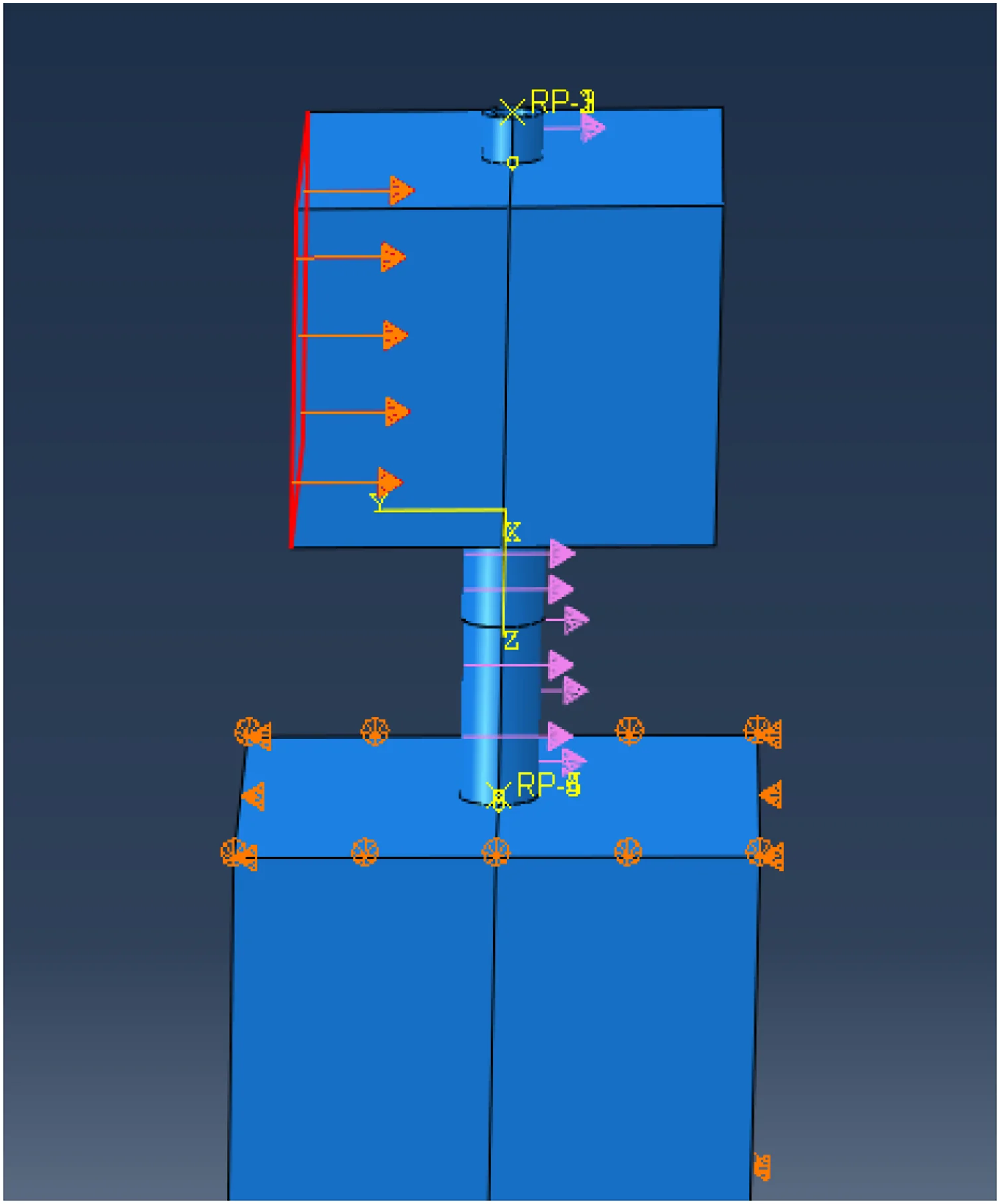
Mechanical model of the Christmas tree in production condition. It is the boundary conditions and loads of the model. The soil model consists of a linear elastic model and a Mohr-Coulomb plastic model, with constraints on soil displacement. The bottom of the conductor is constrained in the X, Y, and Z directions. The outer surface of the conductor is set in surface-to-surface contact with the soil, with normal contact being hard and tangential contact being a penalty function model. Reference points are set at the conductor section at the mud surface, and the section is coupled. The seismic acceleration is applied to the reference points. Current loads are applied along the y-direction to the Christmas tree and high and low pressure wellheads, as uniformly distributed surface forces.

#### 3.3.1 Analysis method for stress state of shallow wellbore.

Establish the force equation for the micro element of the shallow wellbore pipe strings [28], where the lateral force balance equation for the pipe string micro element is:


$$\begin{array}{c} (T+\delta T)sin(\theta +d\theta )+Fcos\theta -(F+\delta F)cos(\theta +d\theta )-Tsin\theta +f_{x}(z,t)\delta z-c\delta z\frac{\partial v(z,t)}{\partial t}=m\delta z\frac{\partial^{\text{2}}v(z,t)}{\partial t^{\text{2}}} \end{array}$$ (5)

The axial force balance equation of the pipe column element is:


$$\begin{array}{c} (T+\delta T)cos(\theta +d\theta )+(F+\delta F)sin(\theta +d\theta )-Tsin\theta -Fsin\theta -f_{z}(z,t)\delta z-w\delta z-c\delta z\frac{\partial u(z,t)}{\partial t}=m\delta z\frac{\partial^{\text{2}}u(z,t)}{\partial t^{\text{2}}} \end{array}$$ (6)

The equation for moment balance of pipe column microelements is:


$$\begin{array}{cc} & (M+\delta M)-M+Tcos\theta dx-Tsin\theta dz+Fcos\theta dz+Fsin\theta dx+((f_{z}(z,t)+w+c\delta z\frac{\partial u(z,t)}{\partial t}\\ & +m\frac{\partial^{\text{2}}u(z,t)}{\partial t^{\text{2}}})\delta z\cdot \frac{dx}{\text{2}}-((f_{x}(z,t)-c\delta z\frac{\partial v(z,t)}{\partial t}+m\frac{\partial^{\text{2}}v(z,t)}{\partial t^{\text{2}}})\delta z\cdot \frac{dx}{\text{2}}=\text{0} \end{array}$$ (7)

Where *T* is the axial tension at the left end of the column element, N; *δT* is axial force increment, N; *θ* is the angle between the tangent line of the axis and the horizontal direction,°; *dθ* is angle increment,°; *F* is lateral shear force at the left end of the pipe column element, N; *δF* is lateral shear force increment, N; f(z,t) is external load distribution of unit length pipe column in the X/Y direction,N/m; *δz* is axial length of tubular microelements,m; *c* is damping coefficient of the pipe column in the x direction, N・s/m²; *v* is axial velocity, m/s; *u* is tangential velocity, m/s; *t* is time, s; *m* is mass per unit length of pipe column, kg; *M* is sec*t*ion bending moment at the left end of the pipe column element,N・m; *δM* is bending moment increment, N・m; w is floating weight per unit length of pipe column, N/m.

#### 3.3.2 Analysis method of wave and current forces.

The calculation of ocean current load can be carried out according to the usual method of calculating resistance [29]. When only ocean currents act, the ocean current load fc per unit length of the structure is:


$$\begin{array}{c} f_{c}=\frac{\text{1}}{\text{2}}C_{D}\rho_{w}Dv_{cmax}^{\text{2}} \end{array}$$ (8)

Where *f*_*c*_ is the current load per unit length, N; $C_{D}$ is the resistance coefficient of seawater, which is related to factors such as Reynolds number Re and surface roughness of the object; $\rho_{w}$ is the density of seawater, kg/m^3^; D is the projected width of the structure facing the current, m; *v*_cmax_ is the maximum possible velocity of the ocean current, m/s。

#### 3.3.3 Soil resistance analysis method.

The ultimate lateral foundation reaction force per unit area on the pipe side can be expressed as [30]:


$$\begin{array}{c} P_{u}=\text{3}C_{u}+\gamma_{c}z+\frac{\varepsilon C_{u}z}{D_{c}},z<z_{r} \end{array}$$ (9)

$$\begin{array}{c} P_{u}=\text{9}C_{u},z>z_{r} \end{array}$$ (10)

$$\begin{array}{c} z_{r}=\frac{\text{6}C_{u}D_{c}}{\gamma_{c}D_{c}+\varepsilon C_{u}} \end{array}$$ (11)

Where $P_{u}$ is the lateral foundation reaction force acting on the pipe side, N; $C_{u}$ is the standard value for undrained shear strength, MPa; $\gamma_{c}$ is bulk density of clay, kg/m^3^; $z_{r}$ is depth of the turning point of lateral foundation reaction force, m; ε is dimensionless constant, generally taken as 0.25 ~ 0.5; $D_{c}$ is outer diameter,m.

## 4. Results and discussion

### 4.1 FMECA analysis of installation and recovery process

#### 4.1.1 Define system boundary.

This article focuses on the testing of whether the components and functions of the Christmas tree are complete during the installation and recovery process, without considering factors in equipment manufacturing, processing, and transportation. In the specific FMECA analysis process, although there are multiple connections of components within the boundary, do not cross the boundary and only analyze the components within the boundary. In addition, the analysis is divided into subsystems based on each component, which are structurally interconnected and functionally interdependent, and are exactly in line with the installation sequence of key components of the Christmas tree.

#### 4.1.2 Divide agreed level.

When conducting risk assessment during the installation and recovery process of the Christmas tree, it is necessary to divide the agreed levels, and the basic principles for dividing the agreed levels depend on the expected results and the availability of design information. In the specific evaluation process, FMECA mainly analyzes the possible failure modes of key components during the installation and recovery process, which determines that the Christmas tree and its installation system are the initial agreed level for FMECA analysis, with the lowest agreed level being the components of the Christmas tree and related tools of the installation system (Fig 6).

**Fig 6:**
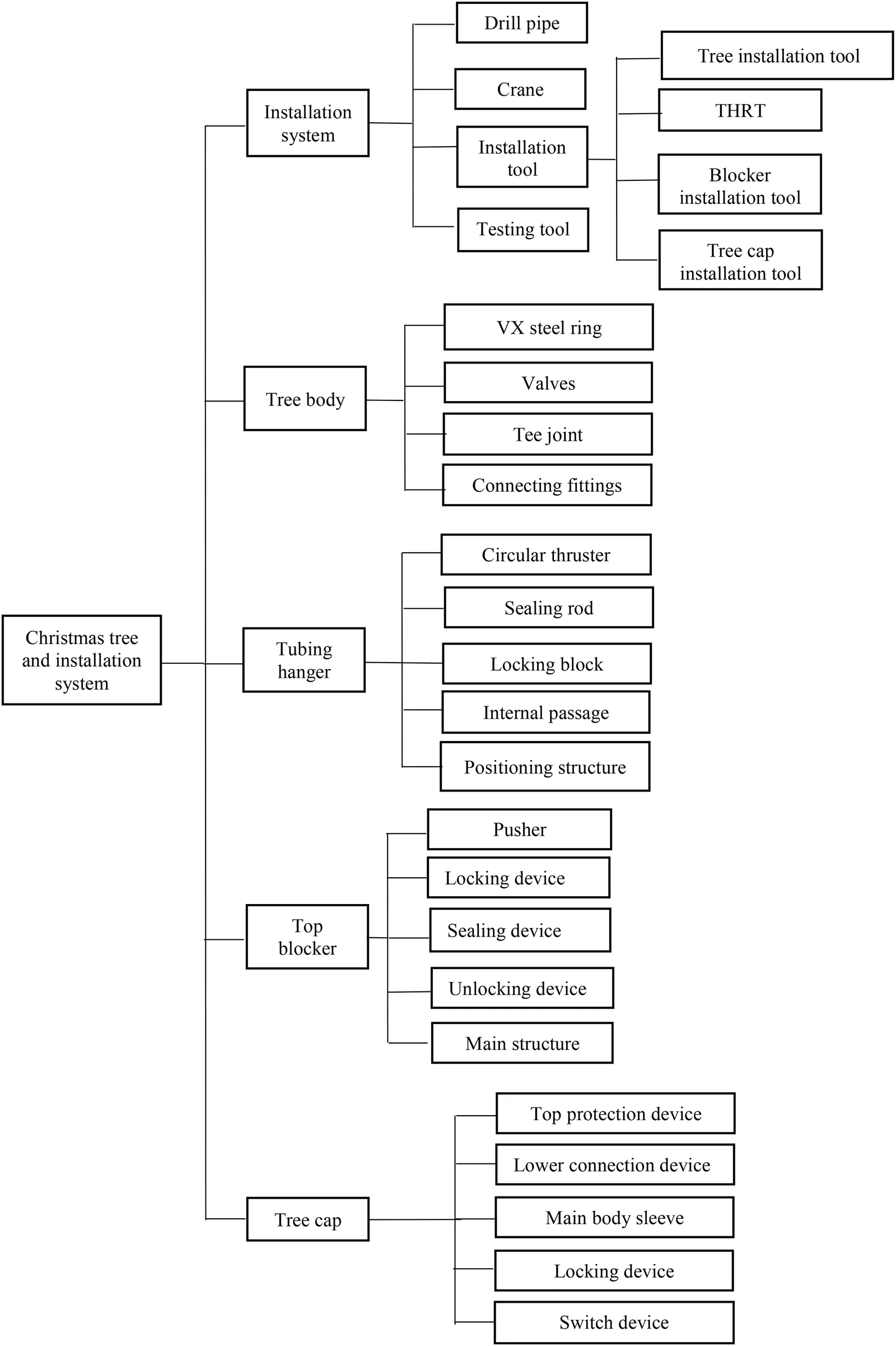
Hierarchical diagram of the Christmas tree system. The Christmas tree and installation system can be divided into several major parts, including the Installation system, Tree body, Tubing hanger, Top blocker, and Tree cap. Each part can be further divided into more detailed sub-levels according to the installation hierarchy.

#### 4.1.3 Fault diagnosis criteria.

The fault criteria should generally be determined based on the specified product functional requirements, corresponding performance parameters, and allowable limits. The fault criteria for the Christmas tree during installation and recovery are as follows:

(1) The Christmas tree cannot be installed successfully.
(2) The installation of the Christmas tree fails the test and is unable to complete the specified function.
(3) The installation and recovery process of the Christmas tree system has caused oil and material losses or impacts on personnel, environment, and other facilities beyond the allowed range.

#### 4.1.4 RPN acceptable standards.

The final RPN value is the product of probability of occurrence, severity, and detectability. The size of the RPN value is a measure of the overall risk of failure modes and an important reference for FMECA analysis results. Therefore, referring to relevant materials, the analysis of RPN values is limited as follows:

(1) RPN > 100, Preventive or improvement measures must be taken.
(2) 100 > RPN > 80, Key considerations, try to avoid as much as possible.
(3) RPN < 80, Consider both factors.

In addition, measures must also be taken for high severity faults with a severity (S) ≥ 8.

#### 4.1.5 FMECA analysis of installation and recovery process of the Christmas tree body.

Transport the Christmas tree to the target location by designated vessel, install and connect the Christmas tree components to the Christmas tree body, conduct tests on land, and inspect the working performance and various indicators of the Christmas tree connection device. After all work indicators are qualified, the Christmas tree body will be lowered. During the lowering process, ROV is used for position tracking, and a heave compensation device is used to control the lowering position of the Christmas tree. Stop lowering when placing it at the target wellhead, use ROV to locate and hydraulically lock the Christmas tree, and ensure it is firmly fixed on the wellhead.

During the installation process of the Christmas tree, all valves are closed. The installation equipment supplies power to the subsea control module through ROV operated electrical and fiber optic connectors, and communicates with the subsea control module. At the same time, the installation equipment provides hydraulic power for the valves and connectors of the Christmas tree. The water installation process monitoring system communicates with the underwater control module to monitor the status information of the installation process in real time. The FMECA for installation and recovery process of Christmas tree body is shown in Table 4.

**Table 4:** FMECA table for installation and recovery process of Christmas tree body.

No.	Name	Function	Failure Mode	Failure reason	Local impact	Ultimate impact	Frequency	Severity	Detection	RPN	Measures
1	Tree body	Provide a closed annular space	1.Overturning 2.Deformation 3. Unable to sit down	1. Excessive sea level winds and waves; Fault in lifting compensation device 2. Uneven force distribution on both sides of the tree body and insufficient strength at the lower threaded connection 3. Excessive vibration; Wave current influence	1. Oil tree detachment 2. Causing the sealing of the tubing hanger or the failure of sealing components such as VX steel rings 3. Causing excessive drift of the work vessel	1. Installation failed 2. Sealing failure, leakage 3. The Christmas tree body cannot be connected to the wellhead	1 2 2	8 6 6	2 3 2	16 36 24	Choose appropriate wind, wave, and current conditions
2	VX steel ring	Sealing of Christmas tree and wellhead	Loosen	1. Loose of threaded connection at the lower part of the Christmas tree body 2. Installation error of VX steel ring	Sealing failure between the wellhead and the tree body causes leakage	Causing leakage in the annular passage, affecting production	2	6	3	36	Using ROV for reinforcement
3	X-over channel	Provide X-over channel connection	1.Leakage 2.Deformation	1. Sealing failure at the connection flange 2. Collision or excessive internal pressure fluctuations	1. Oil and gas leakage during channel conversion operation 2. Causing a decrease in pipeline strength and posing hidden dangers	1. Affects the production process of Christmas tree 2. Causing hidden dangers during the well repair process	3 2	8 6	4 3	96 36	Prioritize conducting onshore sealing test; Operator training
4	BPST vavle	Perform a sealing test between the upper and lower blockers	Leakage	The valve itself or the connecting flange is loose, or the sealing at the flange is ineffective	Sealing performance test between the upper and lower top blockers failed	The non-metallic seal of the upper and lower blockers may fail	2	8	4	64	Prioritize conducting onshore sealing test
5	GTV vavle	VX sealing performance test	Leakage	The valve itself or the connecting flange is loose, or the sealing at the flange is ineffective	Sealing performance test of VX steel ring failed	Sealing failure between wellheads, affecting production	3	8	4	96	Prioritize conducting onshore sealing test
6	Piston	Push the locking clamp to form a sealed chamber	External leakage	Seal failure on the push piston or excessive hydraulic oil pressure	Causing a decrease in pressure within the hydraulic chamber and rendering the locking or unlocking function ineffective	Locking failure, loosening of tree body, causing accidents	3	8	4	96	Add locking indicator alarm device
7	Oil inlet channel	Provide channels to access the hydraulic chamber	1.Leakage 2. Block	1. Loose of hydraulic joint leakage or channel crack leakage 2. Impurity of hydraulic oil generates blockages	1. The locking clamp cannot be tightened, resulting in loose connection 2. Locking or unlocking failed	Loose tree body causes internal sealing failure, affecting production	3 2	8 6	3 3	72 36	1. Optimize connections and verify materials 2. Purified hydraulic oil
8	Oil outlet channel	Provide channels to outflow from the hydraulic chamber	1.Leakage 2. Block	1. Loose of hydraulic joint leakage or channel crack leakage 2. Impurity of hydraulic oil generates blockages	1. The locking clamp cannot be tightened, resulting in loose connection 2. Unlocking failed	1. Loose tree body causes internal sealing failure, affecting production 2. Unable to disassemble	3 2	8 6	3 3	72 36	1. Strengthen connections and materials 2. Purified hydraulic oil
9	Secondary oil inlet channel	Provide channels to access the hydraulic chamber	1.Leakage 2. Block	1. Loose of hydraulic joint leakage or channel crack leakage 2. Impurity of hydraulic oil generates blockages	1. Causing secondary unlocking failure 2. Causing locking or secondary unlocking failure	1. The Christmas tree may not be able to be dismantled 2. Loose tree body affects production, or the Christmas tree cannot be dismantled	3 2	8 6	3 3	72 36	1. Strengthen connections and materials 2. Purified hydraulic oil
10	Secondary outlet channel	Provide channels to outflow from the hydraulic chamber	1.Leakage 2. Block	1. Loose of hydraulic joint leakage or channel crack leakage 2. Impurity of hydraulic oil generates blockages	1. Causing secondary unlocking failure 2. Locking failure	1. Unable to be disassembled 2. Loose tree body, affecting production	3 2	8 6	3 3	72 36	1. Strengthen connections and materials 2. Purified hydraulic oil
11	Action Ring	Provide installation guidance function and support function	1. Loose 2. Crack	1. Loose of threaded connection with the external body 2. Material issues or damage during installation	1. The up and down movement of the connector may cause the hydraulic chamber seal to fail 2. Reduced intensity may cause leakage in the secondary unlocking hydraulic chamber	1. The entire Christmas tree is loose, and internal seals have failed, affecting production 2. Potential hazards to its safety production	2 1	6 8	4 3	48 24	1. Strengthen thread verification 2. Optimize material selection and installation

### 4.2 FTA analysis of production conditions of the Christmas tree

#### 4.2.1 Establishment of module level.

Analyze the production process of Christmas trees and divide them into 11 main system structures, including plugs, tubing hanger, Christmas tree body, wellhead connector, Christmas tree cap, various valves, Christmas tree frames, anti fishing net devices, isolation sleeves, Christmas tree legs, and ROV panels. As an independent system, the fault tree of the Christmas tree can be analyzed as the top event when conducting fault tree analysis. Since the Christmas tree system consists of different modules, fault trees are established for each module for analysis, and then summarized onto the top event of the Christmas tree fault to obtain the fault tree of the Christmas tree system. Based on literature research and statistical analysis of OREDA database, fault trees for 11 main components were established (Figs 7 and 8).

**Fig 7:**
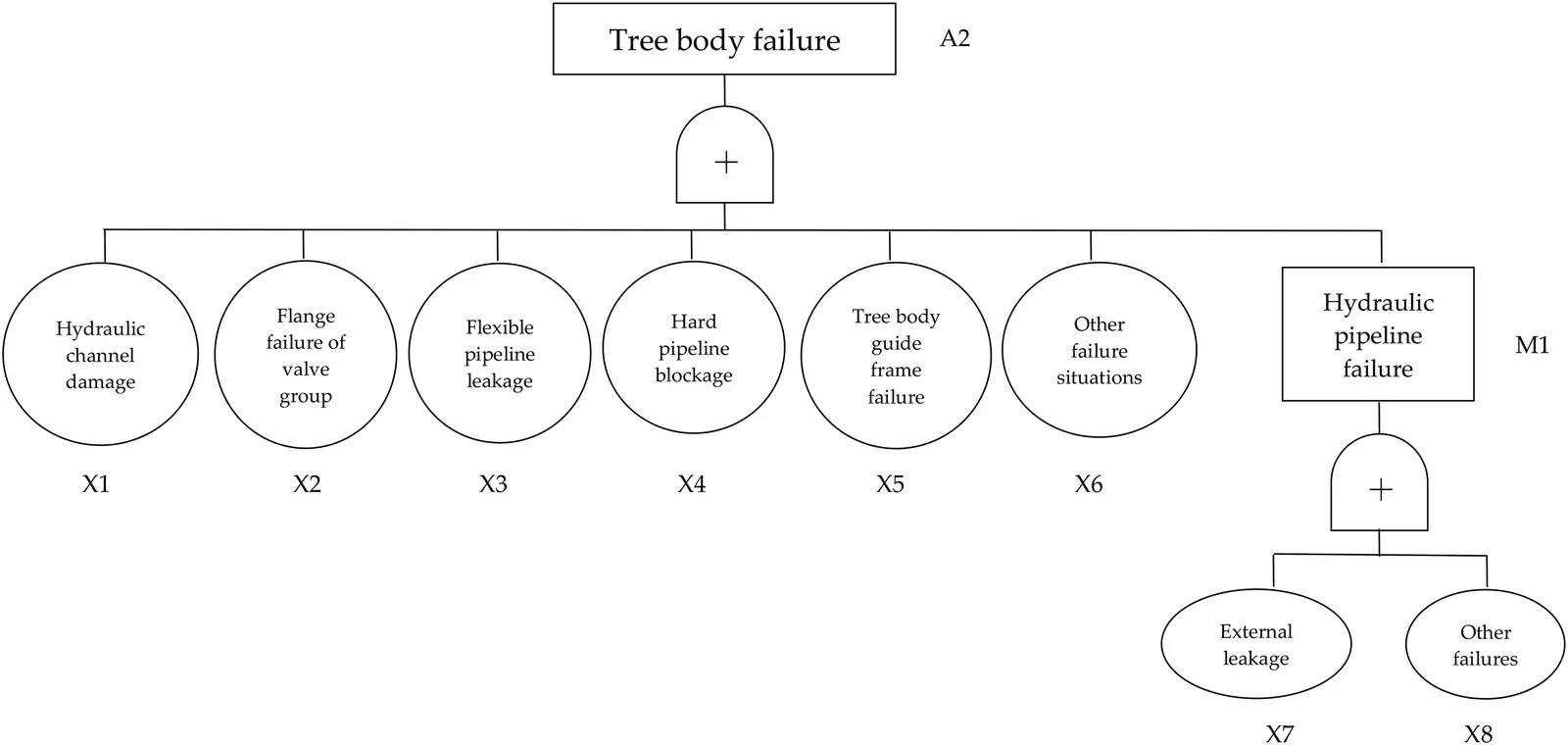
Fault tree of Christmas tree body. According to the tree body failure risk operation procedure, it can be divided into different risk points from X1 to X8, and the description of each risk point is shown in the figure. M indicates the operation risk points that can be further refined.

**Fig 8:**
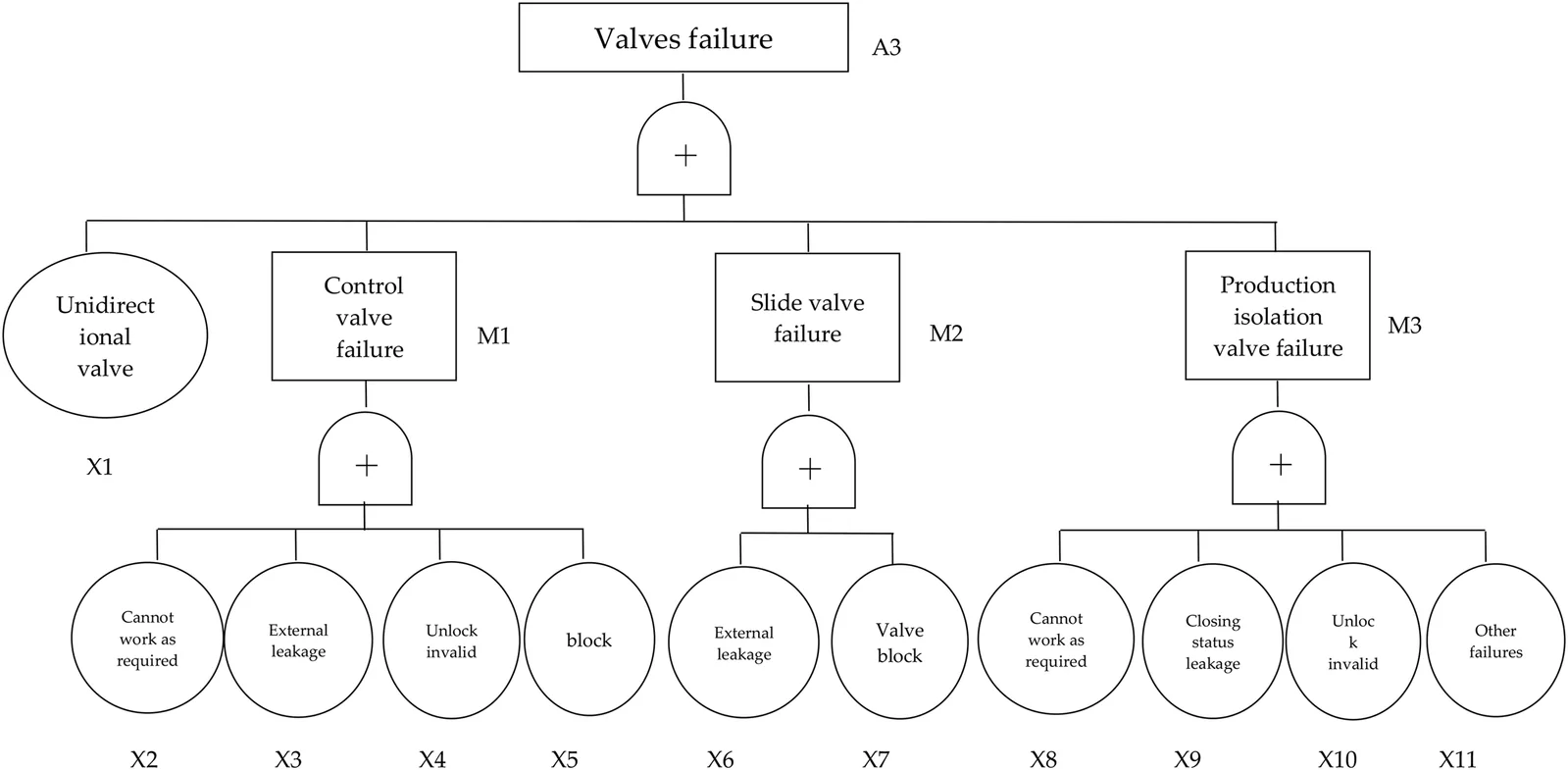
Various fault trees. According to the valve failure risk operation procedure, it can be divided into different risk points from X1 to X11, with the description of each risk point shown in the figure. M indicates the operation risk points that can be further refined.

Taking the fault tree of the Christmas tree as an example, this fault tree has 7 first-order bottom level events and 2 second-order bottom level events. The minimum cut set of the fault tree for the Christmas tree body established based on the fault tree is: {X1},{X2},{X3},{X4},{X5},{X6},{X7},{X8}. In this fault tree, there are a total of 8 minimum cut sets, and each underlying event constitutes a minimum cut set. That is, as long as any underlying event occurs, it will directly lead to the occurrence of the top event. From the fault tree, it can be seen that there are two main types of underlying events that cause the occurrence of the top event: one is blockage caused during operation, and the other is damage caused during daily operation. Therefore, to avoid the occurrence of top incidents, it is necessary to reduce the occurrence of defects during design and installation. At the same time, in daily operations, damage to pipelines should be avoided. Finally, it is necessary to regularly check the working condition of hydraulic pipelines and replace them early to avoid causing greater economic losses.

#### 4.2.2 Probability calculation of each component event.

According to the OREDA fault data manual, the probability of each fault occurring can be calculated. Based on the formula, the probability, importance, and ranking of each basic event can be determined, as shown in the Tables 5–7 below. After calculation, the probability of failure of the Christmas tree body is P = 0.945268, and the importance ranking of the basic event probability of Christmas tree body failure is {X5},{X3},{X4},{X2},{X7},{X1},{X8},{X6}.

**Table 5:** Basic event probability of Christmas tree body.

	X1	X2	X3	X4	X5	X6	X7	X8
P	0.05	0.11	0.53	0.26	0.80	0.4	0.06	0.01

**Table 6:** Probability importance and ranking of Christmas tree body.

	X5	X3	X4	X2	X7	X1	X8	X6
I^Pr^	0.273656	0.116449	0.073961	0.061495	0.058224	0.057611	0.055284	0.054731

**Table 7:** Critical importance and ranking of basic events in tree body.

	X5	X3	X4	X2	X7	X1	X8	X6
C	0.231601	0.065291	0.020343	0.007156	0.003695	0.003047	0.000584	0

#### 4.2.3 Reliability analysis results.

By establishing a fault tree for 11 modules including plugs, tubing hanger, Christmas tree body, wellhead connector, Christmas tree cap, various valves, Christmas tree frames, anti fishing net devices, isolation sleeves, Christmas tree legs, ROV panels, etc., and summarizing the top events of Christmas tree failures, the average failure probability of the Christmas tree system is calculated to be 25.82 times per 10^6^ hours. Therefore, its reliability parameter MTBF (Mean Time Between Failures) is 4.42 years, and the overall reliability level meets the requirements. It is recommended to conduct a systematic maintenance every 4.42 years, using ROV for inspection, with a focus on checking the corrosion status, leakage, and valve operation of the Christmas tree. Combined with sensor parameters such as temperature, pressure and flow rate of the Christmas tree, a comprehensive evaluation should be made to make decisions and carry out corresponding maintenance or repair work.

### 4.3 Stability analysis of Christmas tree in production condition

#### 4.3.1 Stress state analysis.

A well in the South China Sea is selected for stability calculation and analysis of the Christmas tree under operational conditions. The well has a water depth of 234 meters and uses 36 inch low-pressure wellhead and 18–3/4 inch high-pressure wellhead. The top of the high-pressure wellhead has a mud line height of 3 meters. The selected Christmas tree is 8.93 meters long, 8.60 meters wide, 6.45 meters high, and weighs 55 tons. The ultimate bending moment between the wellhead and the Christmas tree is 7797 kN·m. According to the calculation method described in 3.3, the stability of the system under different wind, wave, and current conditions in the well is verified and analyzed. S2 Fig shows the stress conditions experienced by the system under wind, wave, and current conditions that occur once a year, once every ten years, and once every hundred years. According to the operational guidelines, under system connection and non drilling conditions, the ratio of equivalent stress to yield stress of the pipeline is not greater than 0.8. From S2 Fig, it can be seen that the overall maximum stress ratio of the system under the installation condition of the Christmas tree in the well is 0.25, which occurs at the location of the Christmas tree. It can be concluded that the system stability meets the requirements of operational safety under this condition, and there is no risk of installation failure. Similarly, as shown in S3 Fig, when calculating the stress ratio for a 20% offset during drilling platform operation, it can be concluded that the maximum overall stress ratio of the system under this extreme operating condition is 0.529, and there is also no risk of installation failure.

#### 4.3.2 Bending moment calculation analysis.

According to the calculation method described in section 3.3, the system bending moment of the well under different wind, wave, and current conditions is calculated. S4 Fig shows the bending moment experienced by the system under the conditions of once in a year and once in a hundred years wind, wave, and current, and when the displacement of the drilling platform during operation is 20%. Under the conditions of once in a year and once in a hundred years, the maximum bending moment experienced by the system are 5711 kN·m and 7292 kN·m, respectively. According to the ultimate bending moment of 7797 kN·m between the wellhead and the Christmas tree, it can be concluded that there is no risk of production failure in the system, indicating that the pipeline, low-pressure wellhead, high-pressure well, and Christmas tree configuration of the well are reasonable and meet safety production requirements.

#### 4.3.3 Analysis of operation window.

The horizontal and vertical axes of the operation window determine the relationship between the maximum deviation of the drilling platform and the surface current velocity under different operation modes (Fig 9). The allowable deviation of the drilling platform for normal installation and operation under the condition of a once in a year ocean current is 24m in the downstream direction and 28m in the upstream direction, which is less than the distance (46.8m) when the deviation is 20% during drilling platform operation. Therefore, the operating window range calculated by the method in Section 3.3 for this well is safer than the stress and bending moment calculation results.

**Fig 9:**
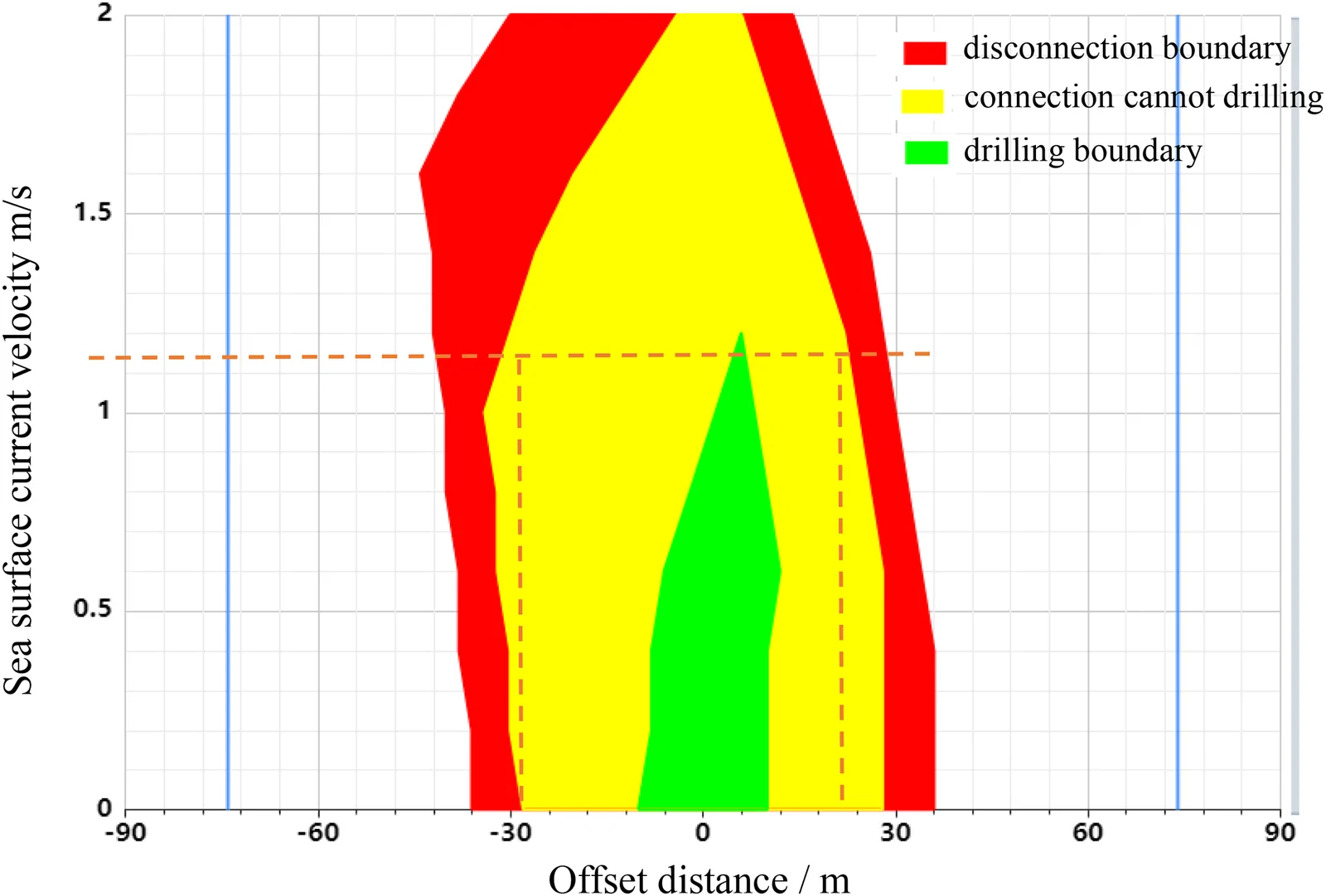
Operation window of the Christmas tree during installation. The green area indicates that normal drilling operations can be carried out, the yellow area indicates that subsea wellhead and Christmas tree installation operations can be carried out, and the red area indicates that the drilling platform has reached the point of disconnection from the Christmas tree and cannot operate normally.

### 4.4 Risk control suggestions

Through FMECA analysis of the installation and recovery process of the Christmas tree, it is evaluated that the main failure modes are deformation of the Christmas tree body, leakage of valve groups, leakage or blockage of connecting pipe fittings. The failure reasons are uneven force on both sides of the tree body, loosening of the valve or connecting flange, loosening and leakage of hydraulic joints or channel cracks. The impact on the system is mainly the overall loosening of the Christmas tree, which causes major accidents and affects production. Further analysis is conducted on the causes and consequences of the accident, identifying the basic or intermediate events that led to the accident. Based on the development process of the accident, potential safety barrier factors are analyzed, and a simplified bow tie model for the installation risk of the Christmas tree is established (Fig 10). It depicts a bow-tie risk model for Christmas tree installation based on FMECA analysis. The model centers around the top event of Christmas tree installation failure, with various failure causes such as body overturning and deformation, valve group leakage, pipeline leakage and blockage, etc., listed on the left side. It is accompanied by multi-level preventive safety barriers such as equipment optimization, sea state selection, onshore pressure testing, monitoring and alarming, personnel training, etc. On the right side, it shows the derivative accident consequences that deteriorate step by step after the barriers fail, including valve body leakage, body deformation, overall loosening of the Christmas tree, and even major offshore production safety accidents. This bow-tie model comprehensively depicts the risk transmission path of “hidden danger causes - core accidents - disaster consequences” in the Christmas tree installation process. It systematically identifies four major risk sources: sea state environment, equipment structural sealing, hydraulic pipelines, and personnel operation, and matches each type of failure with specific engineering control measures. It visually exposes the weaknesses of the existing protection system and provides visual theoretical support for graded risk prevention and control, as well as construction safety plan optimization for subsea Christmas tree installation operations.

**Fig 10:**
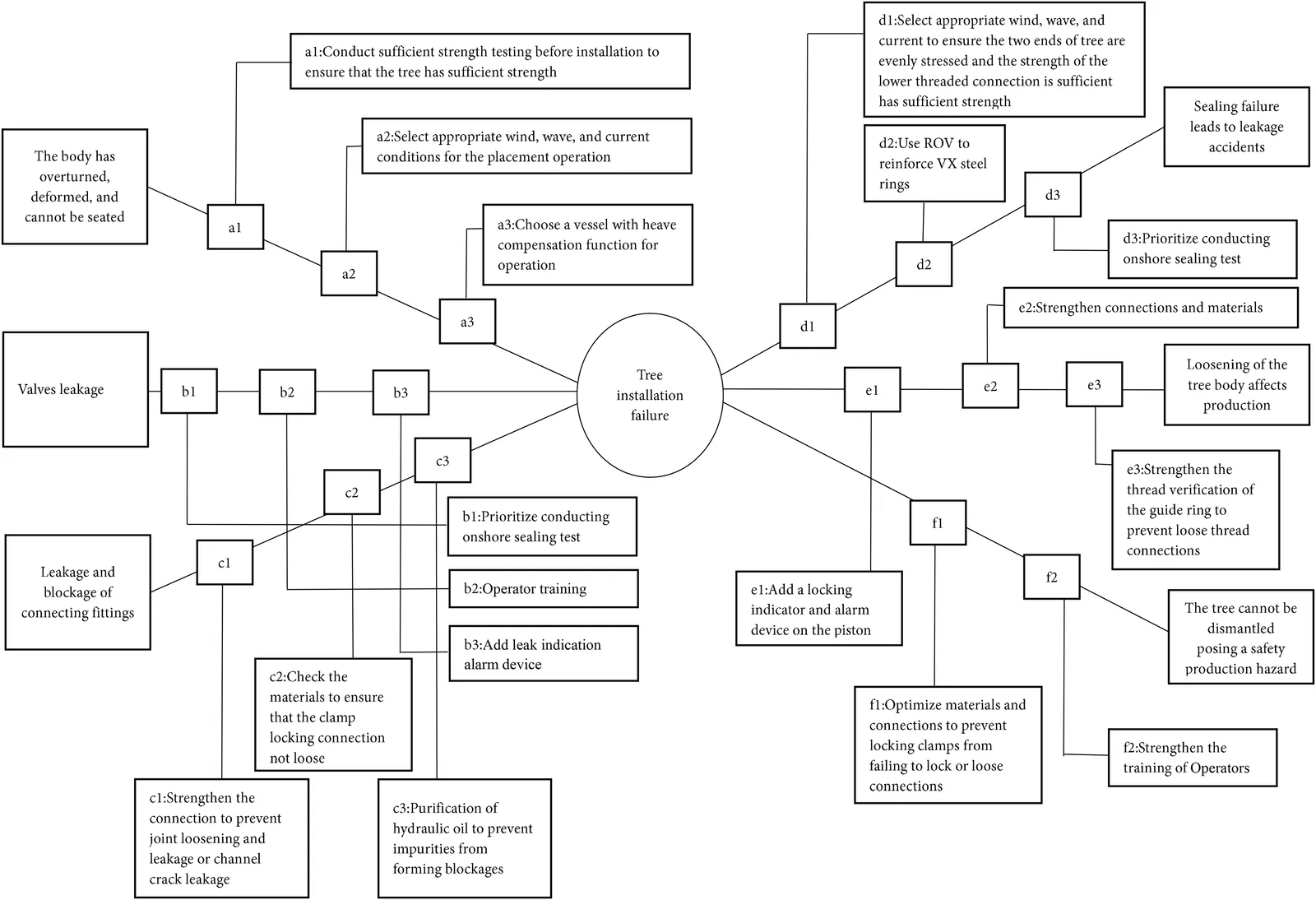
Butterfly bow model of installation risk of the the Christmas body. The model centers around the top event of Christmas tree installation failure, with various failure causes such as body overturning and deformation, valve group leakage, pipeline leakage and blockage, etc., listed on the left side. On the right side, it shows the derivative accident consequences that deteriorate step by step after the barriers fail, including valve body leakage, body deformation, overall loosening of the Christmas tree, and even major offshore production safety accidents.

## 5. Conclusions

This article focuses on the reliability issues of subsea Christmas trees during installation and operation. The significant contribution of this paper lies in proposing a comprehensive method that appropriately integrates Failure Mode, Effects and Criticality Analysis (FMECA), Fault Tree Analysis (FTA), and numerical simulation. The combination of these methods enables the assessment of equipment reliability from multiple perspectives and constructs a comprehensive framework for risk assessment throughout the entire lifecycle of the equipment. The article systematically identifies key failure states of various components, determines their severity levels, and subsequently verifies these states through quantitative analysis using the OREDA database, which is a highly respected operational data source in the field of marine technology. The research results not only provide an evaluation of system reliability but also offer practical suggestions for the safe installation, operation, and maintenance of the equipment. The specific conclusions are as follows:

(1) A survey is conducted on the installation and recovery methods of existing Christmas tree, and the detailed operational steps of the drill pipe installation method are analyzed. Based on the operational experience and installation resources in the South China Sea, the drill pipe installation method is adopted for the installation and recovery of the Christmas tree, with low installation costs, simple operation, and mature technology.
(2) The purpose, principle, and steps of failure mode, effects, and hazard analysis are analyzed, and the FMECA analysis flowchart for the Christmas tree is determined based on the severity and hazard level of failure modes of various components during installation and recovery. The evaluation shows that the main failure modes are deformation of the Christmas tree body, leakage of valve groups, leakage or blockage of connecting pipe fittings. The failure reasons are uneven force on both sides of the tree body, loosening of the valve or connecting flange, loosening and leakage of hydraulic joints or channel cracks. The impact on the system is mainly the overall loosening of the Christmas tree, which may cause major accidents and affect production.
(3) An analysis is conducted on the loads experienced by the Christmas tree under operational conditions. Based on the OREDA database, statistical analysis is conducted on the failure data and related failure modes of the Christmas tree, providing a foundation for FTA analysis of operational conditions of the Christmas tree. A fault tree of 11 main components is established based on the failure of the Christmas tree during production as the top event, and the probability, importance, and ranking of each basic event are calculated. The calculated average failure probability of the Christmas tree system is 25.82 times per 10^6^ hours, therefore its reliability parameter has an average failure interval of 4.42 years, and the overall reliability level meets the requirements.
(4) A numerical simulation analysis is conducted on the overall stability of the Christmas tree system under installation and production conditions. The maximum stress ratio of the system under different ocean current conditions is 0.529, and the maximum bending moment is 7292 kN·m, which meets the overall stress and safety production requirements of the equipment. At the same time, the allowable drilling platform offset for normal installation and operation under one-year ocean current conditions is obtained, and the safety operation window for the installation and production conditions of the underwater Christmas tree system is formulated.
(5) The installation and recovery of the Christmas tree is complex and technically difficult. It is recommended to conduct sufficient strength and sealing performance tests before installation, select suitable operating conditions, strictly follow the installation and recovery process during the operation, strengthen the technical training of operators, and ensure the smooth installation and recovery of the Christmas trees. The safety operation requirements for the Christmas tree during production are high. It is recommended to regularly conduct systematic maintenance and comprehensive evaluation based on reliability analysis results, and carry out corresponding maintenance or repair work.

## Supporting information

S1 Fig Statistics on the frequency of component failures in the Christmas tree. (XLSX)

S2 Fig Maximum stress ratio of the wellhead system (offset = 0). (XLSX)

S3 Fig Maximum stress ratio of the wellhead system (offset = 20%). (XLSX)

S4 Fig Maximum bending moment of the wellhead system. (XLSX)
