# Supplementary material for: A reliability analysis method for Christmas tree in operating conditions based on FEMCA, FTA and numerical simulation
Source: PLoS One. 2026 Oct 8;21(10):e0359208. doi: 10.1371/journal.pone.0359208 (PMC13648976; doi:10.1371/journal.pone.0359208)
Supplement: Peer review history

# Peer review: Decision Letter 0

**DOI:** 10.1371/journal.pone.0359208.r001

**Authors:** Ferdinando Chiacchio

28 Jul 2026

Dear Dr. Wang,

Thank you for submitting your manuscript to PLOS ONE. After careful consideration, we feel that it has merit but does not fully meet PLOS ONE’s publication criteria as it currently stands. Therefore, we invite you to submit a revised version of the manuscript that addresses the points raised during the review process.

Please submit your revised manuscript by Aug 27 2026 11:59PM. If you will need more time than this to complete your revisions, please reply to this message or contact the journal office at plosone@plos.org. When you're ready to submit your revision, log on to https://www.editorialmanager.com/pone/ and select the 'Submissions Needing Revision' folder to locate your manuscript file.

- A letter that responds to each point raised by the academic editor and reviewer(s). You should upload this letter as a separate file labeled 'Response to Reviewers'.
- A marked-up copy of your manuscript that highlights changes made to the original version. You should upload this as a separate file labeled 'Revised Manuscript with Track Changes'.
- An unmarked version of your revised paper without tracked changes. You should upload this as a separate file labeled 'Manuscript'.

If you would like to make changes to your financial disclosure, please include your updated statement in your cover letter. Guidelines for resubmitting your figure files are available below the reviewer comments at the end of this letter.

If applicable, we recommend that you deposit your laboratory protocols in protocols.io to enhance the reproducibility of your results. Protocols.io assigns your protocol its own identifier (DOI) so that it can be cited independently in the future. For instructions see: https://journals.plos.org/plosone/s/submission-guidelines#loc-laboratory-protocols. Additionally, PLOS ONE offers an option for publishing peer-reviewed Lab Protocol articles, which describe protocols hosted on protocols.io. Read more information on sharing protocols at https://plos.org/protocols?utm_medium=editorial-email&utm_source=authorletters&utm_campaign=protocols.

As the corresponding author, your ORCID iD is verified in the submission system and will appear in the published article. PLOS supports the use of ORCID, and we encourage all coauthors to register for an ORCID iD and use it as well. Please encourage your coauthors to verify their ORCID iD within the submission system before final acceptance, as unverified ORCID iDs will not appear in the published article. *Only*  the individual author can complete the verification step; PLOS staff *cannot*  verify ORCID iDs on behalf of authors.

We look forward to receiving your revised manuscript.

Kind regards,

Ferdinando Chiacchio, Ph.D

Academic Editor

PLOS One

Journal Requirements:

When submitting your revision, we need you to address these additional requirements.

1. Please ensure that your manuscript meets PLOS ONE's style requirements, including those for file naming. The PLOS ONE style templates can be found at https://journals.plos.org/plosone/s/file?id=wjVg/PLOSOne_formatting_sample_main_body.pdf and https://journals.plos.org/plosone/s/file?id=ba62/PLOSOne_formatting_sample_title_authors_affiliations.pdf

2. We suggest you thoroughly copyedit your manuscript for language usage, spelling, and grammar. If you do not know anyone who can help you do this, you may wish to consider employing a professional scientific editing service.

The American Journal Experts (AJE) (https://www.aje.com/) is one such service that has extensive experience helping authors meet PLOS guidelines and can provide language editing, translation, manuscript formatting, and figure formatting to ensure your manuscript meets our submission guidelines. Please note that having the manuscript copyedited by AJE or any other editing services does not guarantee selection for peer review or acceptance for publication.

Upon resubmission, please provide the following:

1. The name of the colleague or the details of the professional service that edited your manuscript

2. A copy of your manuscript showing your changes by either highlighting them or using track changes (uploaded as a *supporting information* file)

3. A clean copy of the edited manuscript (uploaded as the new *manuscript* file)

3. Thank you for stating in your Funding Statement:

[This study was supported by the Innovation Project of the Ministry of Industry and Information Technology of China “Engineering Technology Research on Underwater Oil Tree and Its Supporting Tools”(MC-201901-S01-02), Guangdong Provincial Department of Education youth talent project “Research on supply chain management of intelligent construction core construction enterprise” (2023KQNCX128)and the Open Fund of State Key Laboratory of Oil and Gas Reservoir Geology and Exploitation (Southwest Petroleum University) under Grant PLN2022-16.].

Please provide an amended statement that declares *all* the funding or sources of support (whether external or internal to your organization) received during this study, as detailed online in our guide for authors at http://journals.plos.org/plosone/s/submit-now.  Please also include the statement “There was no additional external funding received for this study.” in your updated Funding Statement.

Please include your amended Funding Statement within your cover letter. We will change the online submission form on your behalf.

4. Please remove any funding-related text from the manuscript. Please note that funding information should not appear in any section or other areas of your manuscript. We will publish the funding statement that is present in the Financial Disclosure section of the online submission form.

5. Thank you for stating the following in the Competing Interests section:

[The authors have declared that no competing interests exist.].

We note that one or more of the authors are employed by a commercial company: CNOOC: China National Offshore Oil Corporation.

1. Please provide an amended Funding Statement declaring this commercial affiliation, as well as a statement regarding the Role of Funders in your study. If the funding organization did not play a role in the study design, data collection and analysis, decision to publish, or preparation of the manuscript and only provided financial support in the form of authors' salaries and/or research materials, please review your statements relating to the author contributions, and ensure you have specifically and accurately indicated the role(s) that these authors had in your study. You can update author roles in the Author Contributions section of the online submission form.

Please also include the following statement within your amended Funding Statement.

“The funder provided support in the form of salaries for authors [insert relevant initials], but did not have any additional role in the study design, data collection and analysis, decision to publish, or preparation of the manuscript. The specific roles of these authors are articulated in the ‘author contributions’ section.”

If your commercial affiliation did play a role in your study, please state and explain this role within your updated Funding Statement.

2. Please also provide an updated Competing Interests Statement declaring this commercial affiliation along with any other relevant declarations relating to employment, consultancy, patents, products in development, or marketed products, etc.

Within your Competing Interests Statement, please confirm that this commercial affiliation does not alter your adherence to all PLOS ONE policies on sharing data and materials by including the following statement: "This does not alter our adherence to PLOS ONE policies on sharing data and materials.” (as detailed online in our guide for authors http://journals.plos.org/plosone/s/competing-interests). If this adherence statement is not accurate and there are restrictions on sharing of data and/or materials, please state these. Please note that we cannot proceed with consideration of your article until this information has been declared.

Please include both an updated Funding Statement and Competing Interests Statement in your cover letter. We will change the online submission form on your behalf.

6. In the online submission form, you indicated that [The data that support the findings of this study are available from the corresponding author upon reasonable request.].

All PLOS journals now require all data underlying the findings described in their manuscript to be freely available to other researchers, either 1. In a public repository, 2. Within the manuscript itself, or 3. Uploaded as supplementary information.

This policy applies to all data except where public deposition would breach compliance with the protocol approved by your research ethics board. If your data cannot be made publicly available for ethical or legal reasons (e.g., public availability would compromise patient privacy), please explain your reasons on resubmission and your exemption request will be escalated for approval.

7. We note that Figures 1, 3, 6, 8, and 9 in your submission contain images which may be copyrighted. All PLOS content is published under the Creative Commons Attribution License (CC BY 4.0), which means that the manuscript, images, and Supporting Information files will be freely available online, and any third party is permitted to access, download, copy, distribute, and use these materials in any way, even commercially, with proper attribution. For more information, see our copyright guidelines: http://journals.plos.org/plosone/s/licenses-and-copyright.

We require you to either (1) present written permission from the copyright holder to publish these figures specifically under the CC BY 4.0 license, or (2) remove the figures from your submission:

1. You may seek permission from the original copyright holder of Figures 1, 3, 6, 8, and 9 to publish the content specifically under the CC BY 4.0 license.

We recommend that you contact the original copyright holder with the Content Permission Form (http://journals.plos.org/plosone/s/file?id=7c09/content-permission-form.pdf) and the following text:

“I request permission for the open-access journal PLOS ONE to publish XXX under the Creative Commons Attribution License (CCAL) CC BY 4.0 (http://creativecommons.org/licenses/by/4.0/). Please be aware that this license allows unrestricted use and distribution, even commercially, by third parties. Please reply and provide explicit written permission to publish XXX under a CC BY license and complete the attached form.”

Please upload the completed Content Permission Form or other proof of granted permissions as an "Other" file with your submission.

In the figure caption of the copyrighted figure, please include the following text: “Reprinted from [ref] under a CC BY license, with permission from [name of publisher], original copyright [original copyright year].”

2. If you are unable to obtain permission from the original copyright holder to publish these figures under the CC BY 4.0 license or if the copyright holder’s requirements are incompatible with the CC BY 4.0 license, please either i) remove the figure or ii) supply a replacement figure that complies with the CC BY 4.0 license. Please check copyright information on all replacement figures and update the figure caption with source information. If applicable, please specify in the figure caption text when a figure is similar but not identical to the original image and is therefore for illustrative purposes only.

8. Please amend the Figures 5 and 7 in your manuscript; the current figures are difficult to read.

9. We note you have included a table to which you do not refer in the text of your manuscript. Please ensure that you refer to Table 4 in your text; if accepted, production will need this reference to link the reader to the Table.

If the reviewer comments include a recommendation to cite specific previously published works, please review and evaluate these publications to determine whether they are relevant and should be cited. There is no requirement to cite these works unless the editor has indicated otherwise.

Please review your reference list to ensure that it is complete and correct. If you have cited papers that have been retracted, please include the rationale for doing so in the manuscript text, or remove these references and replace them with relevant current references. Any changes to the reference list should be mentioned in the rebuttal letter that accompanies your revised manuscript. If you need to cite a retracted article, indicate the article’s retracted status in the References list and also include a citation and full reference for the retraction notice.

Additional Editor Comments:

Dear authors.

reviewers have now commented on your submission. They advice with valuable comments on your paper. Therefore in order to proceed with a second re-evaluation, I suggest you to proceed with a review of your manuscript.

Regards

[Note: HTML markup is below. Please do not edit.]

Reviewers' comments:

Reviewer's Responses to Questions

**Comments to the Author**

1. Is the manuscript technically sound, and do the data support the conclusions?

Reviewer #1: Yes

Reviewer #2: Yes

**********

2. Has the statistical analysis been performed appropriately and rigorously?

Reviewer #1: Yes

Reviewer #2: Yes

**********

3. Have the authors made all data underlying the findings in their manuscript fully available?

The PLOS Data policy

Reviewer #1: Yes

Reviewer #2: Yes

**********

4. Is the manuscript presented in an intelligible fashion and written in standard English?

Reviewer #1: Yes

Reviewer #2: Yes

**********

Reviewer #1: The article is devoted to a very interesting and relevant topic. The installation and recovery process of Christmas tree is complex and technically difficult, and it bears high internal and external loads during production and has 16 extremely high requirements for the operation reliability. To ensure the safe produced deepwater subsea Christmas tree in18 stallation and production conditions, a reliability analysis method for Christmas tree in operating conditions based on FEMCA, FTA and numerical simulation is established.

Comments

1. There is scientific novelty in the work, but it needs to be formulated qualitatively in the article.

2. Explanations for Figure 16 and its analysis are needed.

Reviewer #2: The article "A reliability analysis method for Christmas tree in operating conditions based on FEMCA, FTA and numerical simulation" represents a high-quality scientific work focused on the issue of the reliability of a subsea Christmas tree during its installation and operation. The topic is extremely topical given the growing importance of deep-sea hydrocarbon extraction and high requirements for the safety and reliability of subsea production systems. I consider the significant contribution of the article to be a comprehensive methodological approach that appropriately integrates failure mode, effects and criticality analysis (FMECA), fault tree analysis (FTA) and numerical simulations. The combination of these methods allows for the assessment of the reliability of the equipment from multiple perspectives and creates a comprehensive framework for risk assessment throughout the entire life cycle of the equipment. The authors systematically identified critical failure states of individual components, determined their severity and subsequently verified them through quantitative analysis using the OREDA database, which is a respected source of operational data in the field of offshore technologies. The strength of the work is also the connection of theoretical methods with practical application to the first domestic deep-sea subsea Christmas tree in China. The results provide not only an assessment of the system reliability, but also practical recommendations for the safe installation, operation and maintenance of the device. Numerical load simulations under various sea current conditions suitably complement the analytical methods and confirm sufficient strength and stability of the system. Determining a safe operating window represents a significant contribution from the point of view of the real operation of offshore facilities.The conclusion of the article logically follows up on the presented results and provides specific recommendations for operational practice. I particularly appreciate the emphasis on preventive strength and tightness tests, regular maintenance based on the results of the reliability analysis and systematic training of operators. These recommendations increase the practical value of the work and its usability for industrial practice. The article is logically structured, individual methodological steps are clearly described and the results are presented in a clear and understandable manner. The methodology used is scientifically correct, the results are supported by relevant calculations and simulations, and the conclusions adequately reflect the knowledge obtained. Despite the high quality of the work, it would be appropriate to consider supplementing the discussion on the sensitivity analysis of the input parameters used in the FTA model and numerical simulations. Such an analysis would allow a better assessment of the impact of input data uncertainties on the resulting reliability of the system and further support the robustness of the proposed methodology. At the same time, it would be beneficial to briefly compare the obtained results with similar published studies or with other approaches for assessing the reliability of submarine production systems, which would emphasize even more the originality and contribution of the proposed method. The mentioned comments are rather of a recommendatory nature and do not in any way reduce the professional level or the scientific contribution of the submitted article. Overall, I consider the work to be well-crafted, methodically correct.

**********

what does this mean?). If published, this will include your full peer review and any attached files.

If you choose “no”, your identity will remain anonymous but your review may still be made public.

**Do you want your identity to be public for this peer review?**  For information about this choice, including consent withdrawal, please see our Privacy Policy

Reviewer #1: No

Reviewer #2: No

**********

[NOTE: If reviewer comments were submitted as an attachment file, they will be attached to this email and accessible via the submission site. Please log into your account, locate the manuscript record, and check for the action link "View Attachments". If this link does not appear, there are no attachment files.]

To ensure your figures meet our technical requirements, please review our figure guidelines: https://journals.plos.org/plosone/s/figures

You may also use PLOS’s free figure tool, NAAS, to help you prepare publication quality figures: https://journals.plos.org/plosone/s/figures#loc-tools-for-figure-preparation.

NAAS will assess whether your figures meet our technical requirements by comparing each figure against our figure specifications.

# Author comment: Author response to Decision Letter 1

**DOI:** 10.1371/journal.pone.0359208.r002

8 Aug 2026

I have uploaded the Response to Reviewers.docx. You can find the detailed response to the comments there. Thank you.

Attachment Submitted filename: Response to Reviewers.docx

# Peer review: Decision Letter 1

**DOI:** 10.1371/journal.pone.0359208.r003

**Authors:** Ferdinando Chiacchio

10 Sep 2026

A reliability analysis method for Christmas tree in operating conditions based on FEMCA , FTA and numerical simulation

PONE-D-26-24956R1

Dear Dr. Wang,

We’re pleased to inform you that your manuscript has been judged scientifically suitable for publication and will be formally accepted for publication once it meets all outstanding technical requirements.

Within one week, you’ll receive an e-mail detailing the required amendments. When these have been addressed, you’ll receive a formal acceptance letter and your manuscript will be scheduled for publication.

An invoice will be generated when your article is formally accepted. Please note, if your institution has a publishing partnership with PLOS and your article meets the relevant criteria, all or part of your publication costs will be covered. Please make sure your user information is up-to-date by logging into Editorial Manager at Editorial Manager® and clicking the ‘Update My Information' link at the top of the page. For questions related to billing, please contact billing support.

If your institution or institutions have a press office, please notify them about your upcoming paper to help maximize its impact. If they’ll be preparing press materials, please inform our press team as soon as possible -- no later than 48 hours after receiving the formal acceptance. Your manuscript will remain under strict press embargo until 2 pm Eastern Time on the date of publication. For more information, please contact onepress@plos.org.

Kind regards,

Ferdinando Chiacchio, Ph.D

Academic Editor

PLOS One

Additional Editor Comments (optional):

Dear Editors,

reviewers have now commented on your reviewed paper.

Congratulation for the acceptance

Reviewers' comments:

Reviewer's Responses to Questions

**Comments to the Author**

Reviewer #2: All comments have been addressed

**********

2. Is the manuscript technically sound, and do the data support the conclusions?

Reviewer #2: Yes

**********

3. Has the statistical analysis been performed appropriately and rigorously?

Reviewer #2: I Don't Know

**********

4. Have the authors made all data underlying the findings in their manuscript fully available?

The PLOS Data policy

Reviewer #2: Yes

**********

5. Is the manuscript presented in an intelligible fashion and written in standard English?

Reviewer #2: Yes

**********

Reviewer #2: The article "A reliability analysis method for Christmas tree in operating conditions based on FEMCA, FTA and numerical simulation" represents a high-quality and professionally processed scientific work focused on the issue of the reliability of a subsea Christmas tree during its installation and operation. The topic of the work is highly topical given the growing importance of hydrocarbon extraction in the deep sea and the associated high requirements for safety, reliability and operational integrity of subsea production systems. I consider the comprehensive methodological approach, which appropriately integrates the FMECA, FTA and numerical simulation methods, to be a significant contribution of the article. Their mutual connection allows assessing the reliability of the analyzed equipment from multiple perspectives and creates a comprehensive framework for identifying critical failure states, assessing their severity and subsequent quantitative risk assessment. I also positively evaluate the use of data from the OREDA database, which represents a relevant and recognized source of operational data for the field of offshore technologies. The strength of the work is the connection of theoretical methods of reliability analysis with their practical application on a specific subsea production facility. The results are not only of theoretical significance, but also provide practically usable recommendations for the safe installation, operation and maintenance of the facility. Numerical simulations under various sea current conditions suitably complement the analytical part of the work and provide further verification of the strength and stability of the system. The determination of the safe operating window can be considered a significant result in terms of its practical applicability in real offshore operation conditions. I also positively evaluate the final part of the article, which logically follows up on the presented results and formulates specific recommendations for operational practice. The emphasis on preventive strength and leak tests, regular maintenance based on the results of the reliability analysis and systematic training of operating personnel increases the practical significance of the work and its potential contribution to the safe operation of subsea production systems. Compared to the original version, the article has been suitably supplemented and adjusted in areas that were the subject of previous comments. The authors have appropriately expanded the discussion of the sensitivity of the input parameters used in the FTA model and in numerical simulations, thereby better taking into account the impact of input data uncertainty on the analysis results and increasing the robustness of the proposed methodology. The comparison of the obtained results with relevant published studies and approaches used in the assessment of the reliability of subsea production systems has also been appropriately expanded. These modifications have contributed to a better definition of the scientific contribution of the work and at the same time highlighted the originality of the proposed methodological procedure. The article is logically structured, individual methodological steps are clearly presented and the results are adequately discussed. The methods used are scientifically justified and the obtained results are supported by relevant calculations, data and numerical simulations. The conclusions of the work adequately reflect the achieved results and the formulated recommendations have the potential for practical use in the field of design, operation and maintenance of subsea production facilities. Based on the assessment of the modified version of the article, I conclude that the authors have adequately taken into account the comments from the previous review process. The modifications made have increased the scientific quality, completeness and practical value of the work. In my opinion, the article meets the requirements for a scientific publication and makes a relevant contribution to the issue of reliability and safety analysis of subsea production systems.

**********

what does this mean?). If published, this will include your full peer review and any attached files.

If you choose “no”, your identity will remain anonymous but your review may still be made public.

**Do you want your identity to be public for this peer review?**  For information about this choice, including consent withdrawal, please see our Privacy Policy

Reviewer #2: No

**********

# Editor report: Acceptance letter

**DOI:** 10.1371/journal.pone.0359208.r004

**Authors:** Ferdinando Chiacchio

PONE-D-26-24956R1

PLOS One

Dear Dr. Wang,

I'm pleased to inform you that your manuscript has been deemed suitable for publication in PLOS One. Congratulations! Your manuscript is now being handed over to our production team.

At this stage, our production department will prepare your paper for publication. This includes ensuring the following:

* All references, tables, and figures are properly cited

* All relevant supporting information is included in the manuscript submission,

* There are no issues that prevent the paper from being properly typeset

You will receive further instructions from the production team, including instructions on how to review your proof when it is ready. Please keep in mind that we are working through a large volume of accepted articles, so please give us a few days to review your paper and let you know the next and final steps.

Lastly, if your institution or institutions have a press office, please let them know about your upcoming paper now to help maximize its impact. If they'll be preparing press materials, please inform our press team within the next 48 hours. Your manuscript will remain under strict press embargo until 2 pm Eastern Time on the date of publication. For more information, please contact onepress@plos.org.

You will receive an invoice from PLOS for your publication fee after your manuscript has reached the completed accept phase. If you receive an email requesting payment before acceptance or for any other service, this may be a phishing scheme. Learn how to identify phishing emails and protect your accounts at https://explore.plos.org/phishing.

If we can help with anything else, please email us at customercare@plos.org.

Thank you for submitting your work to PLOS One and supporting open access.

Kind regards,

PLOS One Editorial Office Staff

on behalf of

Dr. Ferdinando Chiacchio

Academic Editor

PLOS One
